# Labdane Diterpenoids from *Salvia tingitana* Etl. Synergize with Clindamycin against Methicillin-Resistant *Staphylococcus aureus*

**DOI:** 10.3390/molecules26216681

**Published:** 2021-11-04

**Authors:** Valeria Iobbi, Paola Brun, Giulia Bernabé, Roméo Arago Dougué Kentsop, Giuliana Donadio, Barbara Ruffoni, Paola Fossa, Angela Bisio, Nunziatina De Tommasi

**Affiliations:** 1 Department of Pharmacy, University of Genova, Viale Cembrano 4, 16148 Genova, Italy; valeria.iobbi@edu.unige.it (V.I.); dougue.kentsop.phd@difar.unige.it (R.A.D.K.); paola.fossa@unige.it (P.F.); 2 Department of Molecular Medicine, University of Padova, Via Gabelli 63, 35121 Padova, Italy; paola.brun.1@unipd.it (P.B.); giulia.bernabe@edu.unife.it (G.B.); 3 Consiglio per la Ricerca e la Sperimentazione in Agricoltura–CREA Centro di ricerca Orticoltura e Florovivaismo, 18038 San Remo, Italy; barbara.ruffoni@crea.gov.it; 4 Department of Pharmacy, University of Salerno, Via Giovanni Paolo II 132, 84084 Salerno, Italy; gdonadio@unisa.it (G.D.); detommasi@unisa.it (N.D.T.)

**Keywords:** quorum sensing inhibitors, sclareol, manool, *Salvia tingitana*, molecular docking, callus, pentacyclic triterpenoids

## Abstract

Quorum-sensing (QS) is a regulatory mechanism in bacterial communication, important for pathogenesis control. The search for small molecules active as quorum-sensing inhibitors (QSI) that can synergize with antibiotics is considered a good strategy to counteract the problem of antibiotic resistance. Here the antimicrobial labdane diterpenoids sclareol (**1**) and manool (**2**) extracted from *Salvia tingitana* were considered as potential QSI against methicillin-resistant *Staphylococcus aureus*. Only sclareol showed synergistic activity with clindamycin. The quantification of these compounds by LC–MS analysis in the organs and in the calli of *S. tingitana* showed that sclareol is most abundant in the flower spikes and is produced by calli, while manool is the major labdane of the roots, and is abundant also in the leaves. Other metabolites of the roots were abietane diterpenoids, common in *Salvia* species, and pentacyclic triterpenoids, bearing a γ-lactone moiety, previously undescribed in *Salvia*. Docking simulations suggested that **1** and **2** bind to key residues, involved in direct interactions with DNA. They may prevent accessory gene regulator A (AgrA) binding to DNA or AgrA activation upon phosphorylation, to suppress virulence factor expression. The antimicrobial activity of these two compounds probably achieves preventing upregulation of the accessory gene regulator (agr)-regulated genes.

## 1. Introduction

Antibiotics are mandatory in controlling bacterial diseases both in the community and in healthcare settings. They are one of the most critical medical interventions for surgical procedures, organ transplantations, and management of critically ill subjects, such as patients with cancer. Unfortunately, bacteria have evolved various strategies to avoid or resist the action of antibiotics. While some bacterial species are intrinsically resistant, other bacteria can acquire resistance by the exchange of mobile genetic elements, such as transposons, plasmids, and integrons. The newly acquired elements allow the bacterial cells to modify or destroy the antibiotic, pump the antibiotic out of the cell or decrease the uptake of the molecule, modify, or bypass the target of the antibiotic [1]. Moreover, bacteria can acquire more than one mechanism of antibiotic resistance. Then, the use of antibiotics provides a selective pressure that causes the prevalence of multidrug resistance (MDR) strains and compromises the effectiveness of the drugs [2].

Nowadays, antibiotic resistance is a global problem and antibiotic-resistant pathogens represent a growing risk and burden for human health [3]. The worrisome diffusion of resistant strains and the rapid reduction in the efficacy of antibiotics due to resistance mechanisms or errors in drug administration [4] have led the organizations that monitor human infections, including the Centers for Disease Control and Prevention (CDC), the National Institutes of Health (NIH), the European Centre for Disease Prevention and Control (ECDC), and the World Economic Forum, to estimate that by 2050, infectious diseases caused by resistant pathogens will affect 10 million lives per year and account for 100 trillion dollars of global economic losses [5]. In this scenario, it is essential to target the most harmful bacteria and focus on their resistance mechanisms to develop successful antimicrobial strategies.

*Staphylococcus aureus*, one of the most widespread pathogens in the community and in hospital-acquired infections [6], shows an extraordinary ability to acquire resistance to newly developed antibiotics [7]. Although *S. aureus* is known to be part of the normal skin microbiota, it is frequently the cause of infections involving the respiratory tract, the skin, the soft tissues, and the bloodstream [8]. Over recent years, *S. aureus*-related infections have become more challenging to treat due to an increasing prevalence and diffusion of multi-resistant strains. Indeed, in 2017, 16.9% of hospital acquired infections in Europe were caused by methicillin-resistant *S. aureus* (MRSA) [9,10]. Therefore, antibiotics alone are no longer adequate to control *S. aureus*-induced infections, and there is a need for development of novel strategies to combat the infections caused by MDR strains [6].

Labdane-type diterpenes have been described in the tissues of fungi, insects, marine organisms, and in essential oils, resins, and tissues of higher plants. The main sources of these compounds in the higher plants are the Gymnosperms and Asteraceae, Lamiaceae, and Zingiberaceae [11]. Labdane diterpenoids show several biological activities, such as antimicrobial, antiviral, anti-inflammatory, cytotoxic, antitumor, and phytotoxic effects [11,12,13,14,15]. Two labdanes, namely sclareol and manool, possess bacteriostatic and bactericidal activities [16,17,18,19,20,21] that are of growing interest in controlling antibiotic-resistant pathogens, such as MRSA.

Sclareol (**1**), identified in the aerial parts of *Salvia sclarea* (Lamiaceae) [18,19], is a constituent also of other plant products, such as the resin Ladano from *Cistus creticus* subsp. *creticus* (Cistaceae) [20], the concrete and resinoid of *Nicotiana glutinosa* (Solanaceae) [21,22,23] and the flower and fruit oil of *Cleome spinosa* (Brassicaceae) [24]. This compound is also a starting material for semisynthesis of numerous commercial substances, including production of Ambrox^®^, an ingredient of amber-based fragrances, and related ambergris substitutes used in the formulation of perfumes [25,26,27]. Most commercially produced sclareol is derived from cultivated clary sage (*S. sclarea*); the yield declared by manufacturers when they use solid/liquid extraction (sclareol vs. organic solvent) is approximately 1.5% [28,29].

Manool (**2**) is a minor diterpene of *S. sclarea*, and it is a constituent of other *Salvia* species [19,29,30,31,32]. It has been also isolated from other Lamiaceae sources, i.e., the essential oil of *Origanum scabrum* [33], of *Origanum dayi* [34], and of *Thymus fallax* [35], as well as from other plant families, such as Asteraceae (i.e., *Leuzea acaulis* [36]), Cleomaceae (i.e., *Cleome spinosa* [24], Malvaceae (i.e., *Kitaibelia vitifolia* [37]), Solanaceae (i.e., *Nicotiana longiflora* [38]), and Apiaceae (i.e., *Arctopus* spp. [39]. The commercial natural source of this diterpene is a tree growing in New Zealand, the pink pine (*Halocarpus biformis*, Podocarpaceae), which has the highest known content (up to 6–8%, dry weight) [40].

In a previous study, we reported the presence and the antimicrobial activity of sclareol and manool in the aerial parts *Salvia tingitana* Etl. [41], an aromatic woody-based perennial shrub originating from the Arabian region [42,43] that in the past was considered related to *S. sclarea*, while at present it is supposed a separate species [42,43]. As sclareol is the main constituent (1.2%) of the dichloromethane extract of the aerial parts of *S. tingitana* [41], the present work aimed to investigate the presence of these labdanes also in the total extracts of the aerial parts, the roots, and the flowers of this species, as well as in the callus obtained from in vitro culture, and to assess their activity as quorum-sensing inhibitors (QSI) against MRSA.

Quorum-sensing (QS) is a chemical mechanism of cell-to-cell communication in bacteria that regulates gene expression [44]. This process involves releasing and responding to small signal molecules named autoinducers [45,46]. The concentration of the autoinducers increases as a function of cell density [47]. Quorum-sensing-controlled processes include symbiosis, virulence, competence, conjugation, antibiotic production, motility, sporulation, and biofilm formation [45]. Gram-positive and Gram-negative bacteria use quorum-sensing communication circuits that differ in terms of the nature of the chemical signals, the signal relay mechanisms, and the target-controlled genes [45]. In Gram-positive bacteria, such as in *S. aureus*, the activation of the QS system is mediated by a phosphorylation cascade that influences the activity of DNA-binding transcriptional regulatory proteins called response regulators [44]. The production of small signaling molecules in *S. aureus* is predominantly controlled by the accessory gene regulator (agr) and it has received remarkable attention [48,49]. Agr is also implicated in biofilm formation and dissemination of bacteria [50]. *S. aureus* uses modified oligopeptides as signals and a two-component sensor kinase-response regulator pair, AgrC and AgrA, to communicate [44].

The AgrA response regulator modulates the expression of the gene involved in exotoxin synthesis, and controls the production of virulence factor in *S. aureus* [46,51]. In AgrA receptor, DNA-binding occurs at a site called LytTR domain. This domain is reported to be present in all identified *S. aureus*, including MRSA. Molecular interactions between the LytTR domain and DNA take place in the region of the divergent promoter P2/P3, which controls the transcription of both agr operon and the gene RNAIII/Hld [52]. Alanine mutagenesis and subsequent isothermal titration calorimetry studies have confirmed the importance of residues His169 and Arg233 in AgrA–DNA binding [53]. One of the recent strategies targeting the gene regulation involved in *S. aureus* virulence is the discovery and development of small molecules specifically designed to prevent AgrA binding to DNA [54]

The QS mechanism is thus an interesting target for the development of new anti-infective agents, since it is implicated in various pathologically relevant events. Several studies have shown that inhibition of QS significantly downregulates virulence traits both in vitro and in vivo [54,55,56,57,58]. The selective inhibition of AgrA by itself or in association with antibiotics limits the occurrence of antibiotic-resistant strains and the ability of bacteria to become pathogenic [59].

In the present work we considered the antimicrobial effects of sclareol and manool against MRSA. The antimicrobial activity probably relies on the blocking of the transcriptional function of the agr operon and the prevention of the signal molecule from binding to the response regulator of the agr-regulated genes [60].

## 2. Results

### 2.1. Callus Induction

Callus growth from leaf explants started within 2 weeks, and was influenced by growth regulators and incubation conditions. The calli developed mainly at the level of the leaf central rib in both photoperiod conditions, and presented hard consistency. The percentage of callus formation at different concentrations of plant growth regulators (PGRs) in dark and light conditions after 4 weeks of culture is reported in Table 1.

The highest frequency of callus induction (94.4%) was observed in dark conditions in two media, i.e., the first supplemented with kinetin (KIN) 2.32 µM and 2,4-dichlorophenoxyacetic acid (2,4-D) 2.26 µM, and the second with KIN 4.65 µM and 2,4-D 4.52 µM. The calli obtained in dark conditions were whitish in color and compact (Figure S1, Supplementary Materials). In light conditions, the highest frequency (61.1%) was observed with the combination of KIN 4.65 µM and 2,4-D 4.52 µM. As in dark conditions, no callus formation was observed in the medium lacking 2,4-D. However, at the highest concentration of 2,4-D (22.62 µM), an absence of callus induction was also detected. The calli obtained presented mostly a green whitish color and hard consistency (Figure S2, Supplementary Materials). In both culture conditions, no calli were induced when explants were cultured on the control MS base medium (without plant growth regulator (PGR)) and on all media supplemented only with KIN as PGR. Despite the presence of ascorbic acid, almost all the explants developed dark or brown necrotic areas around the base of the explant in light; yellow or greenish-yellow necrotic areas developed in dark conditions, which in some cases led to explant senescence and death. However, if the browning did not extend over the entire tissue, it apparently did not inhibit callus induction. Induction and callus quality were higher in the dark than in light conditions. For that, calli from dark conditions were selected and maintained in this condition for the subsequent studies.

The viability of calli was verified by fluoresceine diacetate (FDA) staining (Figure S3, Supplementary Materials). The influence of the presence and of the different combinations of PGRs was evaluated, and a significant increase in growth was observed in the presence of PGR, while no significant difference was observed between the various PGR combinations (Figure S4, Supplementary Materials). The culture medium containing the combination of KIN 2.32 µM and 2,4-D 4.52 µM supplemented with ascorbic acid 10 mg/mL was then selected for the biomass production. After several subcultures in this selected medium, the calli became friable (Figure S5, Supplementary Materials). The type of cytokinin, namely KIN, 6-benzylaminopurine (BA) and meta Topolin (mT) (2.32 µM), did not statistically influence the biomass production of callus after 35 days of culture (Figure S6, Supplementary Materials). However, the medium with KIN and mT tended to favor biomass growth in dark condition. Calli were cultured in MS containing KIN 2.32 µM and ascorbic acid 10 mg/L supplemented with equimolar concentration (4.52 µM) of two type of auxin (2,4-D and α-naphthaleneacetic acid, NAA). The maximum average of fresh and dry weights was observed when calli were cultured on a medium containing 2,4-D as auxin with a production of 11.06 ± 2.73 g of fresh or 0.363 ± 0.03 g of dry weight, compared to 3.38 ± 0.74 g of fresh or 0.19 ± 0.03 g of dry weight observed on a medium supplemented with NAA.

**Figure 1:**
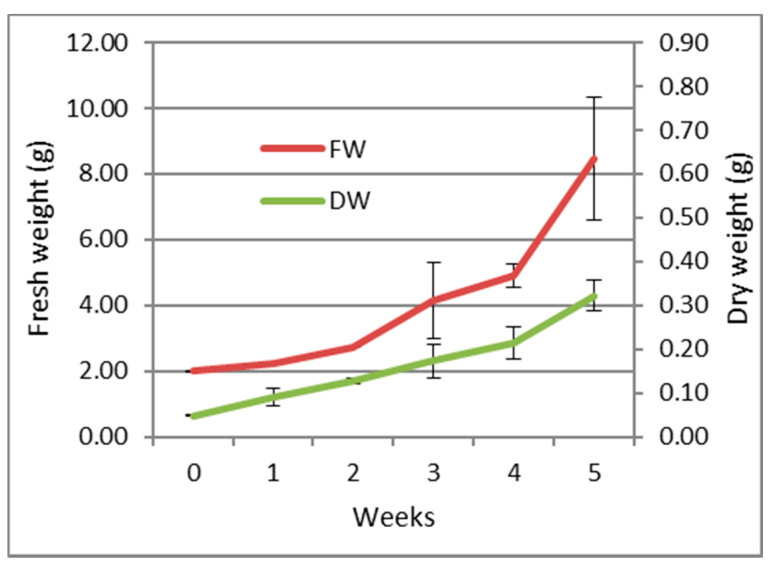
Growth curve of the callus of *S. tingitana* on MS medium supplemented with KIN 2.32 µM and 2,4-D 4.52 µM and ascorbic acid 10 mg/L (average ± SE) *n* = 3. FW: Fresh weight; DW: Dry weight.

The growth parameters (expressed as fresh and dry weight) of the callus over a period of 5 weeks are shown in Figure 1. The callus maintained a slight growth rate during the first four weeks with an exponential increase during the last week reaching a maximum growth.

The biomass production of callus of *S. tingitana* onto 80 Petri capsules in dark conditions (Figure S5, Supplementary Materials) produced 564 g as a final biomass (fresh weight) (26.62 g dry weight).

### 2.2. Phytochemical Analysis

**Figure 2:**
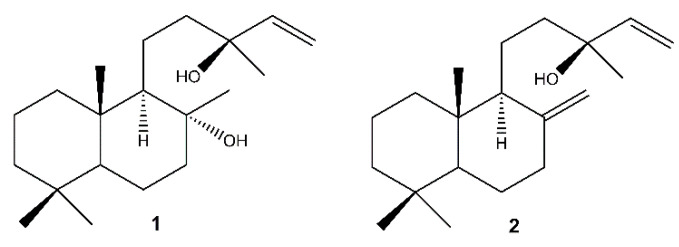
Labdane diterpenoids isolated from *S. tingitana*. **1**: sclareol; **2**: manool.

The chromatographic separation of the methanolic extracts of the roots, the inflorescences and the callus of *S. tingitana* showed the presence of both the labdane diterpenoids sclareol (**1**) [61] and manool (**2**) [62] (Figure 2) in the roots and in the callus, while only **1** was detected in the inflorescences.

**Figure 3:**
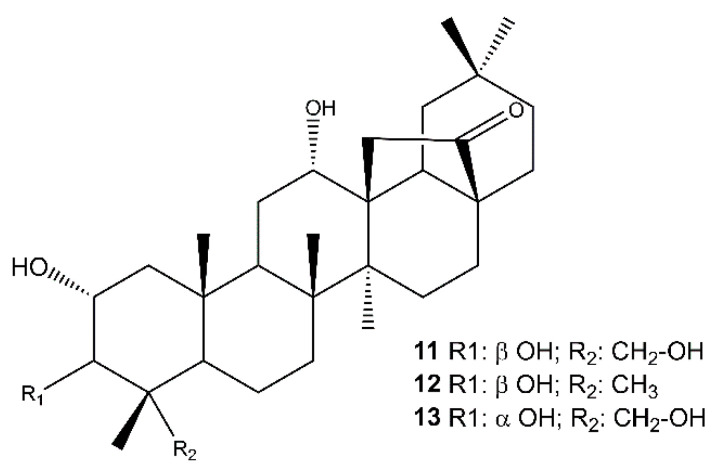
Pentacyclic triterpenoids isolated from the roots of *S. tingitana*.

The analysis of the root extract afforded also the abietanes royleanone (**3**) [63,64] abieta-8,11,13-triene (**4**) [65], cryptojaponol (**5**) [64], abieta-8,11,13-trien-7-one (**6**) [66], ferruginol (**7**) [63], 12-hydroxyabieta-8,11,13-triene-6,7-dione (hypargenin C) (**8**) [67], horminone (**9**) [63,64], 7-*O*-methylhorminone (**10**) [63,68] (Figure S7, Supplementary Materials), and the pentacyclic triterpenoids (Figure 3) 2α,3β,12α,23-tetrahydroxyolean-28,13β-olide (cleistocalyxolide B) (**11**) [69], (2α,3β,12α)-trihydroxy-olean-28,13β-olide (**12**) [70], identified by the comparison of their spectroscopic data with those reported in the literature. The NMR data of compound **13** suggested the same planar structure of compound **12**. The analysis of the chemical shifts and the coupling constants in the ^1^H NMR spectrum (specifically **13**: δ_H_ 3.68 (d, *J* = 3.1 Hz, 1H, C-3), **11**: δ_H_ 3.22 (d, *J* = 9.5 Hz, 1H, C-3) [71,72,73] enabled the definition of **13** as 2α,3α,12α,23-tetrahydroxyolean-28,13β-olide.

The quantification of sclareol (**1**) and manool (**2**) in all the plant extracts, including leaves, flowers, inflorescences, and roots, and in the callus, was achieved using high-resolution LC-MS experiments, and the results are shown in Table 2. Sclareol (**1**) concentrations ranged from 14 to 1241 ppm, with the maximum concentration in the inflorescences, while manool (**2**) was completely absent in the flowers and was more abundant in the root extract.

### 2.3. Quorum Sensing

The sub-inhibitory concentrations of sclareol (**1**) and manool (**2**) against MRSA are reported in Table 3.

The two compounds reported an anti-MRSA activity at concentrations below 32 μg/mL when tested alone. Sclareol reported the lowest antimicrobial concentration.

Synergism assays of the two labdanes with clindamycin were carried by checkerboard assay. Briefly, MRSA cultures were incubated in 96-well plates with serial dilution of clindamycin and **1** or **2** to cross their concentrations. Bacterial growth was recorded 16 h later, and the Fractional Inhibitory Concentration (FIC) was calculated, taking into consideration the subinhibitory concentrations of the compounds and antibiotic. FIC values were considered synergistic when the value was <0.5; antagonistic when the value was >4; additive or indifferent when values were 0.5–4. Our experiments reported that only **1** showed synergistic activity with clindamycin (0.25 μg/mL), with an FIC value of 0.4, while **2** showed an FIC value of 1.1.

### 2.4. Molecular Docking Studies

**Figure 4:**
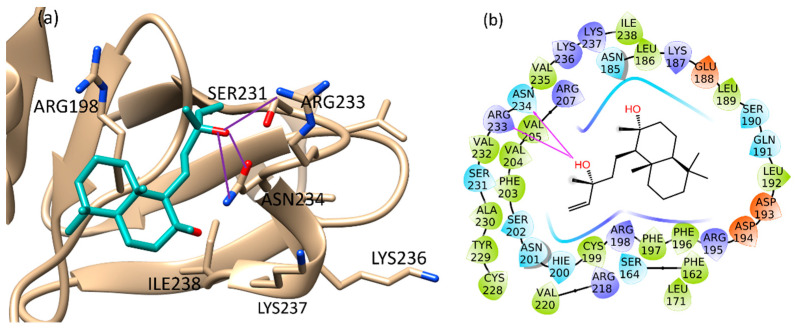
Binding pose (**a**) and interactions (**b**) of sclareol at the conserved AgrA active site. (**a**): the protein is reported as light brown ribbons, sclareol is reported in cyan as capped sticks. H-bonds are presented as purple lines. (**b**): sclareol is surrounded by the protein residues represented as follows: the negatively charged residues are indicated in red, polar residues are in cyan, hydrophobic residues are shown in green. H-bonds are presented as purple arrows.

The binding of sclareol (**1**) and manool (**2**), together with the binding of 4-phenoxyphenol (**A**) and 9H-xanthene-9-carboxylic acid (**B**), described by Leonard et al. [74] as QSI, was evaluated using the Schrödinger Suite 2020–4 [75], focusing the attention on the conserved regions at the C-terminus of AgrA, from Ser231 to Ile238 and also on His200, Agr218, Tyr229, according to literature [76]. In such defined regions, the manool complex was stabilized by a double H-bond interaction with Ile238 and several van der Waals interactions with Ile210 and Ile 238 (Figure S15a, Supplementary Materials). Sclareol (**1**) was bound to Arg233 and Asn234 key residues, which are involved in direct interactions with DNA. In detail, the pentenyl side chain oxygen atom made three H-bonds with Arg233 side chain guanidine moiety, Asn234 backbone nitrogen and side chain carboxamide group (Figure S15b, Supplementary Materials). In addition, the ligand performed hydrophobic interaction with Arg198, close to the Cys199 residue involved in the redox mechanism leading AgrA-DNA interaction (Figure 4) [77].

According to our calculations 4-phenoxyphenol and 9H-xanthene-9-carboxylic acid showed a binding pose in a good agreement with literature data [74]. 4-Phenoxyphenol performed H-bond interactions with its oxygen atoms and residues Arg198 and Asn201, while Arg233 was engaged in a cation-pi interaction with the planar ring of the molecule (Figure S13, Supplementary Materials). The interactions with Arg198 and Asn201 displayed by 4-phenoxyphenol were also confirmed for 9H-xanthene-9-carboxylic acid, which showed an additional H-bond interaction with the key residue Asn234. This predicted binding mode was further stabilized by a salt bridge between the carboxylic acid group of the compound and Arg233 side chain, and by cation–pi interactions with Arg198 and Arg233 (Figure S14, Supplementary Materials). Extra-precision docking mode confirmed the results obtained with the standard procedure (Section 4.6). Sclareol achieved the best binding energy value (−23.294 kcal/mol), followed by 4-phenoxyphenol (−22.191 kcal/mol) and 9H-xanthene-9-carboxylic acid (-20.841 kcal/mol), while manool was scored at −15.747 kcal/mol. Ligand-binding energies and interactions are listed in Table 4.

## 3. Discussion

In the antibiotic-resistance era, the efficacy of any new antimicrobial agents can be quickly limited by the ability of bacteria to rapidly develop resistance to compounds they have never been exposed to before. Among the human bacterial pathogens, *S. aureus* has become resistant to all antibiotics used. The strong ability of *S. aureus* to develop antibiotic resistant strains is linked to the antibiotic mechanism of action and to the virulence of the bacteria. While antibiotics kill or slow down the growth of bacteria, new antibacterial strategies aim to attenuate bacterial virulence to control biofilm formation, sporulation, conjugation, and prevalence of resistant strains. In this scenario, small plant molecules and specifically oxygenated terpenoids can represent important antibacterial agents [78,79], and diterpenoids in particular are reported as good antibiotic enhancers against MRSA [80]. Labdane-type diterpenoids have shown antimicrobial activity against MDR strains [79].

In previous a study, sclareol and manool were isolated from the lipophilic extract of the plant surface of *S. tingitana* [41]. Sclareol, together with similar labdanes, is a constitutive antimicrobial compound of the plant surface [81], and was described in several *Salvia* species (Table S1, Supplementary Materials). The present study showed that sclareol is most abundant in the flower spikes of *S. tingitana*. In *Salvia sclarea*, sclareol is produced mainly (97%) in flower spikes, and specifically in the calyces [82]. Our results showed that in *S. tingitana*, also, there was a considerable gap between the content of sclareol in the flowers and in the inflorescences. Manool is the major labdane of the roots of *S. tingitana*, and it is also abundant in the leaves. The callus of *S. tingitana* could be considered as a source of sclareol that is present at a rate of approximately 8% of that found in the flower spikes, in comparison to the accumulation of this labdane by callus and cell cultures of *S. sclarea*, at rates varying from 0.2 to 6% of those found in the parent plants [83].

The present study also provides a protocol for induction and production of *S. tingitana* calli. The explants of leaves placed on medium without PGRs showed no callus induction; explants of *Salvia leriifolia* had the same behavior [84]. The media supplemented only with 2,4-D showed callus induction in dark or light conditions, justifying the importance of auxin in callus induction [82]. The same findings were reported for *Aquilaria malaccensis* [85]. High concentrations of 2,4-D (22.62 µM) inhibited the induction only in light conditions, suggesting that 2,4-D and the conditions of light or darkness can play a crucial role in the induction of callus of *S. tingitana* [86]. Several investigators have attempted to establish callus cultures from *Salvia* species using a combination of 2,4-D and KIN at various concentrations [87,88]. The selection of suitable combinations of exogenous auxin and cytokinin is a prerequisite to ensure amounts that maintain cell division and expansion. The external supply of plant growth regulators must work in cooperation with endogenous plant hormones to sustain cell growth and development. In this study, the behavior of the *S. tingitana* fresh callus growth curve over time on MS medium supplemented with 2,4-D 4.52 µM, KIN 2,32 µM, and 10 mg/L of ascorbic acid is largely similar to that observed on *S. fruticosa* callus cultures on MS medium supplemented with 4.5 mM 2,4-D and 4.5 mM KIN [89]. The high standard error obtained in this study could be explained by the instability and the variation in vitality of the material between single Petri dishes.

In addition to sclareol (**1**), the chemical analysis of the roots of *S. tingitana* showed the presence of abietane diterpenoids that are common in *Salvia* species [90,91,92,93], and of pentacyciclic triterpenoids (**11**–**13**) characterized by γ-lactone moiety between C-13 and C-17. Compound **11** was previously isolated from *Cleistocalyx operculatus* (Myrtaceae) [69]. (2α,3β,12α)-Trihydroxy-olean-28,13β-olide (**12**) was described by Csuk and co-workers in 2013 [70] as a semi-synthetic 12-*oxo* derivative of oleanolic acid, obtained from lactonization of maslinic acid. To the best of our knowledge, this study represented the first time that this compound was isolated from a plant source. Compared to 2α,3β,12α,23-tetrahydroxyolean-28,13β-olide (**11**), compound **13** showed a different relative configuration at C-2 and C-3, and it is described here for the first time.

The agr quorum-sensing system has been suggested as an emerging and promising antimicrobial drug target, because suppression of the agr system attenuates *S. aureus* virulence in animal models of infection [53,56]. The combination of QSI and antibiotics offers the best therapeutical option because of their multiple targeted actions and low likelihood of resistance [94]. Different extracts from medicinal plants have received increasing attention as effective QS inhibitory substances [95]. Our experimental data showed that sclareol acts synergistically with clindamycin, a protein synthesis inhibitory agent considered as a last therapeutic chance in targeting multi-resistant *S. aureus* strains. However, inducible clindamycin-resistant staphylococcal strains have been isolated more and more frequently in recent years [96]. In our experiments, clindamycin and sclareol were tested at sub-antimicrobial concentrations (Table 3), ruling out the possibility of a direct antibacterial effect. Manool did not show synergistic activity.

With the aim of supporting the experimental data obtained *in vitro*, we investigated the molecular interactions performed by sclareol (**1**) and manool (**2**) with AgrA response regulator, a DNA-binding protein of the agr system [46], by means of a computational study. A careful literature overview highlighted some research studies based on the LytTR domain of the apo AgrA structure from *S. aureus* (PDB code 4G4K) performed by Leonard and co-workers [74], and a previous study (PDB code: 3BS1) [97], which reported the crystal structure of the DNA-binding domain of *S. aureus* AgrA complexed with a DNA pentadecamer duplex. On these bases we evaluated all the PDB structures in the PDB selecting 4G4K [74,76,98,99,100]. Our approach was to evaluate the best resolution of this structure (1.52 Å) among the others, and the different conformations that the protein can assume depending to the presence or the absence of bound DNA. In addition, we thought it fundamental to consider the apo structure of the protein, which was most likely to represent the real target interacting with the compound.

Various studies have reported in silico analysis based on AgrA structure as the target protein of different small molecules expected to bind specifically to the C-LytTR domain and to reduce virulence in *S. aureus*. The relevant goal was to block the interaction between AgrA with P2 and P3 promoters to reduce the virulence of *S. aureus,* with AgrA considered a promising and indispensable target to agrP2- and P3-driven transcription [53]. Several studies focused on His169, Asn201, and Arg233 key residues, which can establish direct interactions with DNA and ligands designed to bind this domain, and may prevent AgrA activation upon phosphorylation and inhibit RNAIII expression and thus the virulence factor deployment. The remarkable role of residues Asn201 and Arg233 in AgrA-mediated QS regulation has been discussed by Parai et al. [99], with emphasis on the uniqueness of the LytTR active site in *S. aureus*. This led us to consider this target useful for developing specific antibiotic therapies for *S. aureus* infections. Several binding inhibitors have already been identified. Daly et al. [76] identified a natural product from the fungus *Penicillium restrictum*, named ω-hydroxyemodin, as a suppressor of QS able to prevent agr signaling in all four *S. aureus* agr alleles. In fact, contrary to AgrC, which has four variants in *S. aureus*, AgrA displays the same sequence in all four subgroups, potentially emerging as an optimal therapeutic target [101]. The most favorable binding site for *ω*-hydroxyemodin was found near the AgrA–DNA interface in a pocket between the side chains of residues His200, Agr218, Tyr229, and Val232, recently identified as major contributors to maximal AgrA activity [102]. Another recent study reported *ω*-hydroxyemodin able to inhibit virulence activation across all four agr subtypes [103]. In contrast to savirin, *ω*-hydroxyemodin was also observed to inhibit agr-system activation in *S. epidermidis*. Savirin also binds the AgrA of *S. aureus*, interacting with Tyr229, adjacent to Cys228 (a residue critical for AgrA folding), and Arg218 near the DNA binding interface. Finally, morin was reported to interact with DNA. This compound binds the target in a conserved region located between Ser231 and Ile238, where it forms four hydrogen bonds [98]. This pocket has the peculiarity of exhibiting 100% identity among 211 Staphylococcal strains, as evidenced by Leonard and co-workers [74]. Finally, Palaniappan et al. [55] highlighted the binding of bumetanide in this region, in particular the interaction with the conserved Tyr-229 of AgrA, and underlined how inhibiting AgrA would be a practical approach to inhibit/reduce virulence, making the pathogen more vulnerable to the host immune system and antibiotic therapy. This study has the merit to evaluate in silico also the ligands studied by Leonard [74]: 4-phenoxyphenol (compound A), 9H-xanthene-9-carboxylic acid (compound B), 2-(4-methylphenyl)-1,3-thiazole-4-carboxylic acid (compound C), and savirin, as previously described. In this docking study, sclareol and manool demonstrated interesting interactions with the studied targets and a positive alignment with 4-phenoxyphenol (compound A), 9H-xanthene-9-carboxylic acid (compound B). These compounds were found to fit well with AgrA protein, interacting with the amino acid residues (Arg198, Asn201, Ile210, Ile213, Arg233, Asn234, Ile238), which are responsible for AgrA-mediated QS regulation. Sclareol and manool showed better binding energies than 4-phenoxyphenol and 9H-xanthene-9-carboxylic acid, supporting the hypothesis of their potential inhibitory activity of bacterial quorum sensing. According to microbiological data, sclareol reveals a higher binding energy in the receptor pocket than manool.

Our data support the idea that natural plant compounds can interfere with bacterial regulator systems. It could be supposed that the formation of the AgrA–sclareol complex leads to the disabling of AgrA signal receptors. Since the AgrA signaling pathway serves as a transcriptional activator of virulence factors in *S. aureus*, we conclude that sclareol (**1**) synergize with clindamycin to block AgrA phosphorylation and the bacterial ability to cause infection and disease. Moreover, by preventing the binding of AgrA with DNA or by avoiding AgrA activation, sclareol could inhibit RNAIII expression and production of the downstream virulence factor [53]. This finding agrees with the idea that the association of multiple compounds is effective in inhibiting the quorum-sensing systems in bacteria, resulting in multi-target therapy for the treatment of bacterial infections [94].

## 4. Materials and Methods

### 4.1. General Experimental Procedures

NMR experiments were performed on a Bruker DRX-600 spectrometer (Bruker BioSpin GmBH, Rheinstetten, Germany) equipped with a Bruker 5 mm TCI CryoProbe at 300 K and a Bruker DRX-400 spectrometer. All 2D NMR spectra were acquired in CDCl_3_, and standard pulse sequences and phase cycling were used for TOCSY, COSY, ROESY, NOESY, HSQC, and HMBC spectra. The NMR data were processed using UXNMR software. The ROESY spectra were acquired with tmix = 400 ms. HRESIMS data were acquired in the positive ion mode by an LTQ Orbitrap XL mass spectrometer (Thermo Fisher Scientific, San Jose, CA, USA). The Orbitrap mass analyzer was calibrated according to the manufacturer’s directions using a mixture of caffeine, methionine-arginine-phenylalanine-alanine-acetate (MRFA), sodium dodecyl sulfate, sodium taurocholate, and Ultramark 1621. Data were collected and analyzed using the software provided by the manufacturer. MPLC chromatography was performed on a Spot Liquid Chromatography system (Armen Instrument, Saint Ave, France) with Normal Phase Si60 Cartridges Supervarioflash and LiChroprep RP-18 (40–63 μm) (Merck, Darmstadt, Germany). Silica gel 60 F254-coated aluminum sheets (Merck, 20 × 20 cm, 0.2 mm layer thickness) were used for TLC. CHCl_3_-CH_3_OH-HCOOH (10:0.5:0.1) was used as mobile phase, and spots were detected by spraying with 50% H_2_SO_4_, followed by heating. Semi-preparative HPLC were carried out using a Waters W600 pump equipped with a Rheodyne Delta 600 injector, a 2414 refractive index detector, and a 2998 photodiode array detector (all Waters Corporation, Milford, MA, USA). A C18 column, SymmetryPrep C18, 7.8 × 300 mm ID, 7 µm particle size (Waters) was used at room temperature, flow rate 2.0 mL/min, sample loop 100 µL, eluents A: H_2_O, B: CH_3_OH, gradient: 5% to100% B in 61 min, 100% B to 75 min.

### 4.2. Plant Material

The fresh aerial parts, the inflorescences, the flowers, and the roots of a commercial specimen of *S. tingitana* were obtained from CREA OF in San Remo, Italy. The plant material was identified by Prof. Ammar Bader, and a voucher specimen (UQU-IT-2019/1) was deposed in the Laboratory of Pharmacognosy at Umm Al-Qura University, Saudi Arabia.

### 4.3. Callus Induction

#### 4.3.1. Callus Initiation

Leaf explants were taken from a mother plant growing in the greenhouse of CREA OF in San Remo, Italy. Leaves from the second and the third node were gently excised, and then first washed with tap water for 15 min and then with soapy water for 15 min, followed by a treatment with 1% of active chlorine supplemented with some drops of Tween 20 for 15 min. Explants were finally rinsed three times with sterile distilled water for 10 min each. After sterilization, the leaves were cut along the midrib and the fragments (1 to 1.5 cm in length) were inoculated onto different culture media. All types of culture media consisted of agarized Murashige and Skoog (MS) medium [104] added with ascorbic acid 10 mg/L [88,89] to reduce medium oxidation and explant tissues necrosis, supplemented with different combinations of KIN and 2,4-D (Table 5).

The media were adjusted to pH 5.7 ± 0.2 using NaOH or HCl, the agar was then added (0.8% of plant agar). The media were autoclaved at 121 °C and 1 atm for 20 min and poured into polystyrene Petri dishes, 90 mm diameter (25 mL of medium/dish). For each medium, three Petri dishes containing 6 leaf explants were prepared and sealed with Parafilm. Two cultural conditions were investigated: light conditions with a photoperiod of 16 h of light at 30 μE m*−2*s*−1*, and 8 h of dark or dark conditions 24/24. The experiment was carried out for 4 weeks at 23 ± 2 °C. After this period, quantity and quality data were recorded. The frequency of callus induction was calculated according to the following formula:


(1) $$\mathrm{Callus}\;\mathrm{induction}\;\mathrm{frequency}\;(\%)=\frac{\mathrm{No}.\;\mathrm{of}\;\mathrm{explants}\;\mathrm{producingcallus}\;}{\mathrm{No}.\;\mathrm{of}\;\mathrm{explants}}\;\times \;100$$

After these 4 weeks, a sample part of the newformed callus was transferred to the respective culture medium without 2,4-D in the same cultural conditions for possible development of somatic embryos.

#### 4.3.2. Callus Viability

The viability test was performed using fluorescein diacetate (FDA). The stock solution of FDA was prepared by diluting FDA in acetone (5 mg/mL) and stored at −18 °C. Immediately before staining, a sample of this solution was diluted 100 times with distilled water to make the final solution (50 μg/mL) and laid over the fresh material. Living callus was immersed in a drop of this solution for 30 min in dark condition. The material was mounted on the microscopic glass slides and observed with the fluorescence microscopy (LEICA DM 4000 B with GFB filter cube: excitation range blue, excitation filter BP 470/40, dichromatic mirror 500, suppression filter BP525/50) and the pictures were taken with LEICA DFC 350 FX.

#### 4.3.3. Influence of Growth Regulators on Callus Biomass Production

Three concentrations of KIN (0.46; 2.32 and 4.65µM) in combination with 2,4-D (2.25 and 4.53 µM) and medium without hormone “MS0” as a control (Table 5) were used. All media were supplemented with ascorbic acid 10 mg/L. Six Petri dishes were prepared for each combination, and the fresh and dry weights were evaluated after 35 days of culture only in dark conditions and at a temperature of 23 ± 2 °C. In a further step, three cytokinins at equimolar level (2.32 μM), namely kinetin, benzyl adenine, and meta-Topolin, were combined with 2,4-D 4.52 μM supplemented with ascorbic acid 10 mg/L. In other to find the best auxin, kinetin 2.32 μM was combined with an equimolar concentration (4.52 μM) of 2,4-D or NAA, supplemented with ascorbic acid 10 mg/L. Four Petri-dishes were prepared for each combination. The fresh and dry weights were evaluated after 35 days of culture in dark conditions and at a temperature of 23 ± 2 °C.

#### 4.3.4. Growth Kinetic

To develop a growth curve of the callus, 2 g pieces of fresh callus were inoculated onto 25 mL solid MS medium supplemented with KIN 2.32 µM, 2,4-D 4.52 µM and 10 mg/L of ascorbic acid named MC medium (MC) in darkness and at 23 ± 2 °C. Eighteen Petri dishes were prepared. Every one-week interval up to five weeks, three Petri-dishes were randomly chosen and analyzed for fresh and dry weight.

#### 4.3.5. Callus Biomass Production

The callus was then cultured onto Petri dishes containing 25 mL of MC medium. The medium was changed every 5 weeks for 14 months to reach a large biomass amount. The final biomass yield was obtained by culturing 80 Petri dishes containing the same medium composition and initially charged with approximately 3.55 g of fresh callus into 25 mL of medium. The cultures were incubated in the growth chamber at 23 ± 2 °C in darkness for 5 weeks. The total biomass was harvested, and the fresh and dry weights were determined.

### 4.4. Phytochemical Analysis

#### 4.4.1. Extraction of the Plant Material

The fresh aerial parts (516.9 g), the inflorescences (154.0 g), the flowers (158.7 g), the roots (209.0 g), and the dried callus (26.62 g) of *S. tingitana* were thoroughly extracted with methanol, affording 8.3 g, 2.5 g, 9.2 g, 24.0 g, and 7.5 g of ground extracts, respectively.

#### 4.4.2. Analysis of the Methanolic Extracts

The extracts of the inflorescences were fractionated by Si gel MPLC (Merck Kiesegel 60, 230–400 mesh, 200 g) (Merck, Darmstadt, Germany) eluting with *n*-hexane/CHCl_3_/CH_3_OH at concentrations varying from 100:0:0 to 0:0:100 (1.7 L), to obtain 15 fractions. Fraction 6 (30.5 mg) (eluted with CHCl_3_ from 0.84 to 0.99 L) was purified by semi-preparative RP HPLC, affording a mixture of ursolic and oleanolic acids and **1** (1.2 mg).

The extract of the roots was fractionated by Si gel MPLC (Merck Kiesegel 60, 230–400 mesh, 200 g) (Merck, Darmstadt, Germany) eluting with *n*-hexane/CHCl_3_/CH_3_OH at concentrations varying from 100:0:0 to 0:0:100 (1.7 L) to obtain 11 fractions. Fraction 2 (87.6 mg) (eluted with *n*-hexane/CHCl_3_ from 0.15 to 0.33 L) was purified by semi-preparative RP HPLC, affording **3** (1.2 mg) and **4** (5.2 mg). Fraction 3 (310.1 mg) (eluted with CHCl_3_ from 0.33 to 0.36 L) was purified by semi-preparative RP HPLC, affording **5** (3.5 mg), **6** (3.5 mg), and **7** (11.0 mg). Fraction 4 (730.0 mg) (eluted with CHCl_3_ from 0.36 to 0.39 L) was purified by semi-preparative RP HPLC, affording **6** (1.5 mg), **8** (2.4 mg), **9** (2.0 mg), and **3** (2.0 mg). Fraction 5 (770.7 mg) (eluted with CHCl_3_ from 0.39 to 0.42 L) was purified by semi-preparative RP HPLC, affording **8** (1.8 mg), **7** (2.4 mg), **6** (3.1 mg) and **2** (5.4 mg). Fraction 6 (120.7 mg) (eluted with CHCl_3_ from 0.42 to 0.54 L) was purified by semi-preparative RP HPLC, affording **10** (2.3 mg) and **1** (1.2 mg). Fraction 7 (183.7 mg) (eluted with CHCl_3_ from 0.54 to 1.14 L) was purified by semi-preparative RP HPLC, affording **11** (2.1 mg). Fraction 8 (167.7 mg) (eluted with CHCl_3_/CH_3_OH from 1.14 to 1.23 L) was purified by semi-preparative RP HPLC as above, affording **12** (1.8 mg) and **13** (2.0 mg).

The extract of the callus was fractionated by Si gel MPLC (Merck Kiesegel 60, 230–400 mesh, 200 g) (Merck, Darmstadt, Germany) eluting with *n*-hexane/CHCl_3_/CH_3_OH at concentrations varying from 100:0:0 to 0:0:100 (1.7 L) to obtain 13 fractions. Fraction 4 (40.5 mg) (eluted with CHCl_3_ from 0.48 to 0.72 L) was purified by semi-preparative RP HPLC, affording **2** (2.3 mg). Fraction **6** (40.5 mg) (eluted with CHCl_3_ from 0.96 to 1.14 L) was purified by semi-preparative RP HPLC, affording a mixture of ursolic and oleanolic acids and **1** (1.2 mg).

#### 4.4.3. 2α,3α,12α,23-Tetrahydroxyolean-28,13β-olide (13)

^1^H NMR (600 MHz, CDCl_3_): δ = 4.20 (br s, 1H, CH (12)), 4.02 (m, 1H, CH (2)), 3.68 (d, *J* = 3.1 Hz, 1H, CH (3)), 3.51 ^a^ (1H, CH2 (23)), 3.49 ^a^ (1H, CH_2_ (23)), 2.35 (m, 2H, CH_2_ (21)), 2.30 (m, 1H, CH_2_ (11)), 2.17 ^a^ (1H, CH_2_, (19)), 2.16 ^a^ (1H, CH_2_, (16)), 2.03 ^a^ (1H, CH_2_, (19)), 2.02 ^a^ (1H, CH, (18)), 1.93 ^a^ (1H, CH_2_, (15)), 1.91 ^a^ (1H, CH, (9)), 1.79 ^a^ (1H, CH_2_, (11)), 1.74 ^a^ (1H, CH_2_, (1)), 1.70 ^a^ (1H, CH, (5)), 1.67 ^a^ (1H, CH_2_, (7)), 1.63 ^a^ (2H, CH_2_, (22)), 1.43 ^a^ (1H, CH_2_, (6)), 1.41 ^a^ (1H, CH_2_, (6)), 1.42 (s, 3H, CH_3_, (27)), 1.36 (m, 1H, CH_2_, (21)), 1.31 ^a^ (1H, CH_2_, (1)), 1.29 ^a^ (1H, CH_2_, (7)), 1.28 ^a^ (1H, CH_2_, (16)), 1.26 ^a^ (1H, CH_2_, (15)), 1.20 (s, 3H, CH_3_, (26)), 1.00 (s, 3H, CH_3_, (29)), 0.96 (s, 3H, CH_3_, (25)), 0.90 (s, 3H, CH_3_, (30)), 0.71 (s, 3H, CH_3_, (24)) ppm. ^13^C NMR (150 MHz, CDCl_3_): δ = 175.9 (C=O, C28), 92.2 (C-O, C13), 79.2 (CHOH, C3), 71.7 (CH_2_OH, C23), 66.9 (CHOH, C2), 65.2 (CHOH, C12), 52.4 (CH, C18), 46.2 (C, C17), 45.0 (CH, C9), 44.5 (C, C14), 42.9 (C, C8), 42.8 (CH, C5), 41.9 (CH_2_, C1), 41.5 (C, C4), 40.2 (CH_2_, C19), 38.2 (C, C10), 34.4 (CH_2_, C7), 33.8 (CH_2_, C21), 33.7 (CH_3_, C29), 32.5 (C, C20), 29.9 (CH_2_, C11), 29.5 (CH_2_, C15), 25.3 (CH_2_, C22), 24.1 (CH_3_, C30), 21.8 (CH_2_, C16), 20.7 (CH_3_, C27), 19.3 (CH_3_, C26), 18.6 (CH_3_, C25), 17.7 (CH_2_, C6), 17.6 (CH_3_, C24) ppm (^a^ = overlapped signals; assignments were confirmed by COSY, 1D-TOCSY, HSQC and HMBC experiments). HRESIMS *m*/*z* 505.3512 [M + H]^+^ (calcd. for C_30_H_49_O_6_ 505.3529)

#### 4.4.4. Determination of the Content of Sclareol (**1**) and Manool (**2**)

High-resolution LC-MS analyses were carried out to quantify sclareol and manool in the different extracts, using an LTQ-orbitrap spectrometer coupled with an Accela UHPLC (Thermo Fisher Scientific). Pure compounds were used to set up and validate the method. Mass spectra were acquired in positive high-resolution single ion monitoring (hrSIM) mode, to maximize selectivity and sensitivity. Ions 291.2685 [sclareol-H_2_O + H]^+^ and 273.2582 [manool-H_2_O + H]^+^ were monitored for the two compounds. Chromatography was performed on a Kinetex C8 column (100 × 2.1 mm, 1.7 µm; Phenomenex) using a mixture of 0.1% formic acid in water (Eluent A) and methanol (Eluent B) as mobile phase. Compound elution was achieved through a gradient from 55 % to 85 % of B over 7 min. Using this method, a Lower Limit of Detection (LLOD) of 0.1 µg/mL was measured for both compounds, whereas the Lower Limit of Quantization (LLOQ) was 0.2 µg/mL, and the response was linear over a 0.3–6 µg/mL concentration range (Figure S12, Supplementary material). Different samples were analyzed in triplicate, injecting 10 µL of each 1 mg/mL extract.

### 4.5. Quorum-Sensing Experiment

#### 4.5.1. Bacterial Strain and Growth Conditions

Methicillin-resistant *S. aureus* (MRSA; ATCC33592) was purchased from ATCC (LGC Standards; Milan, Italy). MRSA was maintained in Lysogeny broth (LB.; Fisher Scientific; Milan, Italy). The day before the experiment, MRSA cultures were inoculated in fresh LB (dilution 1:100) and grew for 16 h at 37 °C.

#### 4.5.2. Antimicrobial Susceptibility Profiles

Before the synergism assays, the minimal inhibitory concentrations (MIC) of the extracts and the elected antibiotic clindamycin were determined for MRSA. Cultures of MRSA were collected, centrifuged, and dispensed in 96-well microtiter plates at 1 × 10^6^ CFU/well final concentrations. Plant extracts were added to bacterial cultures at final concentrations ranging from 0 to 200 μg/mL; clindamycin was added at final concentrations ranging from 0 to 2 μg/mL. The plates were incubated at 37 °C under continuous shaking for 24 h. Bacterial growth was quantified 24 h later by measuring the optical density at 620 nm. The concentrations (MIC) that inhibited MRSA growth were recorded, and the MIC 90% was calculated. One-fourth of the MIC 90% was considered as the subinhibitory concentration [105] of the plant extracts or clindamycin, and used in the synergism assays. Data were confirmed by plating the bacterial cultures on LB agar plates; growth colonies were enumerated. All the experiments were repeated at least three times with duplicate determinations for each condition.

#### 4.5.3. Synergistic Activity of Plant Extracts and Clindamycin

To check the synergy of plant extracts with clindamycin, we set up checkerboard assays to calculate Fractional Inhibitory Concentration (FIC) values as previously described by Rabadia et al. [106]. MRSA cultures (10^6^ CFU/well) were added to 96-well plates where clindamycin was serially diluted along the ordinate, and plant extracts were diluted along the abscissa. Plates were incubated for 16 h at 37 °C, and the optical density was measured to evaluate bacterial growth. The ΣFIC values were calculated as follows:

ΣFIC= FIC_A_ + FIC_B_

where FIC_A_ is the MIC of drug A (clindamycin) in the combination/MIC of drug A (Clindamycin) alone, and FIC_B_ is the MIC of drug B (plant compound) in the combination/MIC of drug B (plant compound) alone. FIC values were considered as follows [17]: Synergy <0.5; Antagonism >4; Additive or indifference 0.5–4. All the experiments were repeated three times with single determinations for each condition.

### 4.6. Molecular Docking

#### 4.6.1. Protein Preparation

The crystal structure of *S. aureus* AgrA LytTR domain (PDB code: 4G4K) [74] was obtained from the Protein Data Bank [107]. Missing side chains and hydrogens were added and optimized using the Protein Preparation Wizard embedded in Schrödinger Suite 2020 [75], and pH was set to 6.0 ± 1.0 value, optimizing the protonation states and the formation of disulfide bridges. Water molecules were removed according to the protocol already described by Sastry et al. [108]. The structure was then energy-minimized using the OPLSe3 forcefield to constrain heavy atoms. The binding site of AgrA was defined by us as previously described by Leonard et al., as a common locus at the C-terminal end of the LytTR domain, a site known to be important for DNA binding activity [74]. The Receptor Grid Generation tool in the Glide module [109] was used to set up a grid that allowed the prepared ligands to bind into the receptor pocket [108]. The box was defined as a region centered at the active site (Ser231, Val232, Arg233, Asn234, Lys236, Lys237 and Ile238) where compounds were anchored.

#### 4.6.2. Ligand Preparation

The chemical structures of sclareol (**1**), manool (**2**), 4-phenoxyphenol [74] and 9H-xanthene-9-carboxylic acid [74] were built with Maestro Build Panel [75] and energetically minimized with LigPrep module [110] using OPLS3e force field [111]. For each ligand, a conformational search was performed considering all possible tautomers and protonation states at a pH of 6.0 ± 1.0. The generated conformers were then clustered by means of the Clustering of conformer tool, and the lowest energy conformer from each cluster for each ligand was considered for docking studies.

#### 4.6.3. Docking Studies

A 20 × 20 × 20 Å^3^ grid box was centered at the active site defined by Leonard et al. [74], from residue Ser231 to Ile238 and by using a search box large enough to include also His200, Arg218, and Tyr229. The Glide-SP and the Glide-XP flexible docking approaches were consecutively applied [109] using the Schrödinger Suite 2020–4 [75]. For each ligand the two approaches resulted in agreement, proposing similar binding modes. The interaction pattern of the selected ligand molecules was analyzed by the Ligand interaction diagram Maestro’s tool [75] and detected with the Protein–Ligand Interaction Profiler (PLIP) tool [112].

### 4.7. Statistical Analysis

Data were analyzed using the R environment [113]. All experiments were conducted using a completely randomized design. The analysis of variance was conducted using the *aov* function (from the statistical package). The treatment levels were compared for significant differences using Tukey’s test (*p* < 0.05) with the function Tukey HSD. The data are presented as means ± standard error.

## 5. Conclusions

Many of the virulence factors produced by pathogenic bacteria depend on quorum sensing, a microbial communication system. By interfering with quorum sensing, small molecules can disarm the virulence of bacteria, alone or in combination with traditional antibiotics. The final aim is to reduce antibiotic concentration and avoid the occurrence of antibiotic resistance. In this study, we reported that the therapeutic combination of an antimicrobial agent with a low molecular weight plant product, such as labdane derivatives, can display promising effects [80] and that diterpenoids can act as adjuvants for antimicrobials exhibiting synergy effects. The computational finding agreed with the experimental data, since only sclareol showed synergistic activity with clindamycin and higher energy value in binding to the receptor pocket, in comparison with manool. To our knowledge, this is the first study of the establishment of callus cultures of *S. tingitana* involving chemical analysis of the roots of this species; moreover, one of the lactonized pentacyclic triterpenoids isolated from the roots is reported here for the first time.

## Figures and Tables

**Table 1:** Callus induction from leaf of *S. tingitana* on MS medium supplemented with different levels and combinations of PGRs (KIN: 0, 0.46, 2.32, 4.65 and 2,4-D: 0, 2.26, 4.52, 22.62 µM) in light and dark conditions *^a^*.

MS + Ascorbic Acid 10 mg/L + PGRs (µM)		Photoperiod (h of Light)			
		16 h		0 h	
KIN	2,4-D	% Induction	Callus Behavior and Quality *^b^*	% Induction	Callus Behavior and Quality *^b^*
0	0	0	-	0	-
0	2.26	11.1	Whitish green, *	55.6	Whitish, *
0	4.52	16.7	Whitish green, *	38.9	Whitish, *
0	22.62	0	-	5.6	Whitish, *
0.46	0	0	-	0	-
0.46	2.26	44.4	Whitish green, **	83.3	Whitish, ***
0.46	4.52	16.7	Whitish green, **	77.8	Whitish, **
0.46	22.62	0	-	33.3	Whitish, *
2.32	0	0	-	0	-
2.32	2.26	22.2	Brown, **	94.4	Whitish, ***
2.32	4.52	27.8	Brown, **	50	Whitish, **
2.32	22.62	0	-	22.2	Whitish, *
4.65	0	0	-	0	-
4.65	2.26	33.3	Whitish green, **	77.8	Whitish, ***
4.65	4.52	61.1	Whitish green, ***	94.4	Whitish, **
4.65	22.62	0	-	44.4	Whitish, *

*^a^* Data reported after 28 days; PGRs: Plant Growth Regulators; *^b^* Quality of callus: * lowquality, ** medium quality, *** high quality; KIN: kinetin, 2,4-D: 2,4-dichlorophenoxyacetic acid.

**Table 2:** Concentration (ppm) of sclareol (**1**) and manool (**2**) in the methanolic extracts of leaves, flowers, inflorescences, roots, and callus of *S. tingitana*.

Plant Biomass	Sclareol (1)	Manool (2)
Leaves	14	329
Flowers	15	ND
Inflorescences	1241	ND
Roots	72	1644
Callus	97	19

**Table 3:** Sub-inhibitory concentrations (μg/mL) of clindamycin and sclareol (**1**) and manool (**2**).

Stimulus	Sub-Inhibitory Concentration
Clindamycin	0.25
**1**	70.0
**2**	81.5

MRSA cultures were incubated with different concentrations of **1** and **2** (range 0–200 µg/mL) or clindamycin (range 0 to 2 μg/mL). Bacterial growth was quantified 24 h later by measuring the optical density at 620 nm. One-fourth of the MIC 90% was considered as the sub-inhibitory concentrations.

**Table 4:** Docking interaction parameters of the studied compounds.

Ligand Molecules	Glide Binding Energy (kcal/mol)	H-Bond Interacting Amino Acids	Hydrophobic Interactions
Sclareol	−23.294	Arg233, Asn234	Arg198
Manool	−15.747	Ile238	Ile210, Ile213
4-Phenoxyphenol	−22.191	Arg198, Asn201	Arg233
9H-Xanthene-9-carboxylic acid	−20.841	Asn201, Asn234	Arg198, Asn201, Arg233

**Table 5:** Combinations of growth regulators (µM) used to induce callus from leaf explants of *S. tingitana ^a^*.

		2,4-D (µM)			
		0	2.26	4.52	22.62
**KIN** **(µM)**	**0**	0; 0	0; 2.26	0; 4.52	0; 22.62
	**0.46**	0.46; 0	0.46; 2.26	0.46; 4.52	0.46; 22.62
	**2.32**	2.32; 0	2.32; 2.26	2.32; 4.52	2.32; 22.62
	**4.65**	4.65; 0	4.65; 2.26	4.65; 4.52	4.65; 22.62

*^a^* KIN: kinetin, 2,4-D: 2,4-dichlorophenoxyacetic acid.

## Data Availability

The data presented in this study are available upon request from the corresponding author.

## Supplementary Materials

The following are available online. Figure S1. Callus of *S. tingitana* developed from leaf explants in dark conditions on MS supplemented with different combinations of PGRs and 10 mg/L of ascorbic acid after 4 weeks. Figure S2. Callus of *S. tingitana* developed from leaf explants in light conditions on MS supplemented with different combination of PGRs and 10 mg/L of ascorbic acid after 4 weeks. Figure S3. FDA staining of *S. tingitana* callus. Figure S4. Effects of different combinations of plant growth regulators on *S. tingitana* callus biomass production. Figure S5. Callus of *S. tingitana*. Figure S6. Effects of equimolar concentrations of different cytokinins on callus growth. Figure S7. Abietane diterpenoids isolated from the roots of *S. tingitana*. Figure S8. ^1^H NMR (600 MHz, CDCl_3_) spectrum of compound **13**. Figure S9. HSQC (600 MHz, CDCl_3_) spectrum of compound **13**. Figure S10. HMBC (600 MHz, CDCl_3_) spectrum of compound **13**. Figure S11. COSY (600 MHz, CDCl_3_) spectrum of compound **13**. Figure S12. Calibration curves obtained for the LC/MS/MS analysis of sclareol (**1**) and manool (**2**) using pure compounds. Figure S13. Binding poses and ligand interaction plots of 4-phenoxyphenol to the conserved AgrA active site. Figure S14. Binding poses and ligand interaction plots of 9H-xanthene-9-carboxylic acid to the conserved AgrA active site. Figure S15. Superimposition of sclareol, 4-phenoxyphenol and 9H-xanthene-9-carboxylic acid in the AgrA binding cavity. Figure S16. Manool and sclareol interactions with the key residues into the binding cavity. References from [114,115,116,117,118,119,120,121,122,123,124,125,126,127,128,129,130,131,132,133,134,135,136,137,138,139,140,141,142,143,144,145,146,147,148,149,150,151,152,153,154,155,156] are cited from the supplementary materials.

## Sample Availability

Samples of the compounds are available from the authors.
