# Supplementary material for: Labdane Diterpenoids from Salvia tingitana Etl. Synergize with Clindamycin against Methicillin-Resistant Staphylococcus aureus
Source: Molecules. 2021 Nov 4;26(21):6681. doi: 10.3390/molecules26216681 (PMC8587691; doi:10.3390/molecules26216681)
Supplement: Supplementary file 1 [file molecules-26-06681-s001.zip › molecules-1431066-supplementary.pdf]

molecules

Supplementary Material for:

Labdane Diterpenoids from *Salvia tingitana* Etl. Synergize with Clindamycin against Methicillin-Resistant *Staphylococcus aureus*

Valeria Iobbi [1], Paola Brun [2], Giulia Bernabé [2], Roméo Arago Dougué Kentsop [1,3], Giuliana Donadio [4], Barbara Ruffoni [3], Paola Fossa [1], Angela Bisio[1,*], and Nunziatina De Tommasi [4]

[1] Department of Pharmacy, University of Genova, Viale Cembrano 4, 16148 Genova, Italy; valeria.iobbi@edu.unige.it (V.I.); paola.fossa@unige.it (P.F.); dougue.kentsop.phd@difar.unige.it (R.A.D.K.);
[2] Department of Molecular Medicine, University of Padova, Via Gabelli 63, 35121, Padova, Italy; paola.brun.1@unipd.it (P.B.); giulia.bernabe@edu.unife.it (G.B.)
[3] Consiglio per la Ricerca e la Sperimentazione in Agricoltura–CREA Centro di ricerca Orticoltura e Florovivaismo, 18038, San Remo (IM), Italy; barbara.ruffoni@crea.gov.it
[4] Department of Pharmacy, University of Salerno, Via Giovanni Paolo II 132, 84084 Salerno, Italy; gdonadio@unisa.it (G.D.), detommasi@unisa.it (N.D.T.)
* Correspondence: bisio@difar.unige.it

CONTENT:

Figure S1. Callus of *S. tingitana* developed from leaf explants in dark condition on MS supplemented with different combination of PGRs and 10 mg/L of ascorbic acid after 4 weeks.
Figure S2. Callus of *S. tingitana* developed from leaf explants in light condition on MS supplemented with different combination of PGRs and 10 mg/L of ascorbic acid after 4 weeks.
Figure S3. FDA staining of *S. tingitana* callus.
Figure S4. Effect of different combination of plant growth regulators to *S. tingitana* callus biomass production.
Figure S5. Callus of *S. tingitana*.
Figure S6. Effect of equimolar concentration of different cytokinins on callus growth.
Figure S7. Abietane diterpenoids isolated from the roots of *S. tingitana.*
Figure S8. ^{1}H NMR (600 MHz, $CDCl_3$) spectrum of compound **13**.
Figure S9. HSQC (600 MHz, $CDCl_3$) spectrum of compound **13**.
Figure S10. HMBC (600 MHz, $CDCl_3$) spectrum of compound **13**.
Figure S11. COSY (600 MHz, $CDCl_3$) spectrum of compound **13**.
Figure S12. Calibration curves obtained for the LC/MS/MS analysis of sclareol (**1**) and manool (**2**) using pure compounds.
Figure S13. Binding pose and ligand interaction plot of 4-phenoxyphenol at the conserved AgrA active site.
Figure S14. Binding pose and ligand interaction plot of 9H-xanthene-9-carboxylic acid at the conserved AgrA active site.
Figure S15. Manool and sclareol interactions with the key residues into the binding cavity.

Table S1. Literature survey about the presence of sclareol (**1**) and manool (**2**) in *Salvia* spp.[a] 10

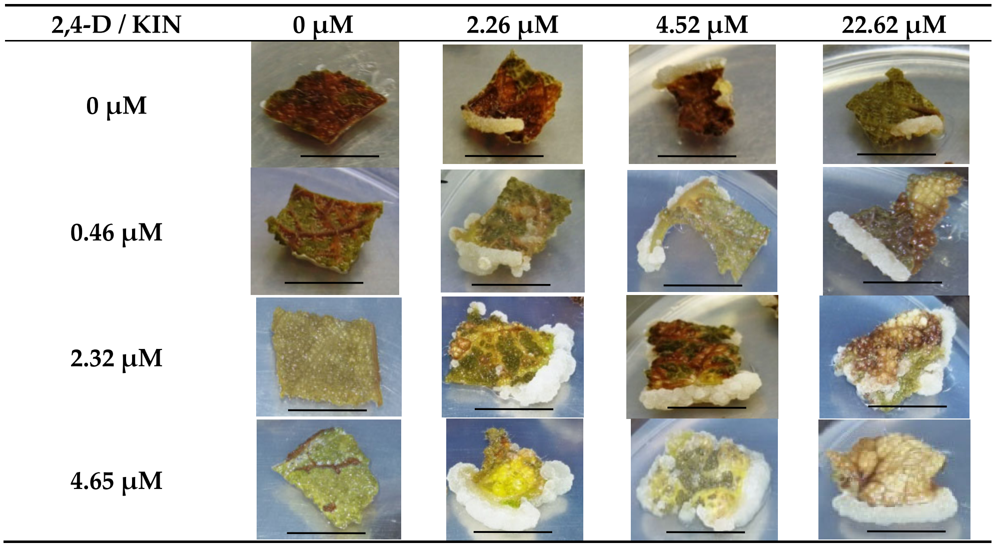

Figure S1. Callus of *S. tingitana* developed from leaf explants in dark condition on MS supplemented with different combination of PGRs and 10 mg/L of ascorbic acid after 4 weeks.MS: Murashige and Skoog medium; PGRs: plant growth regulators; 2,4-D: 2,4-dichlorophenoxyacetic acid; KIN: kinetin. Bars = 1 cm.

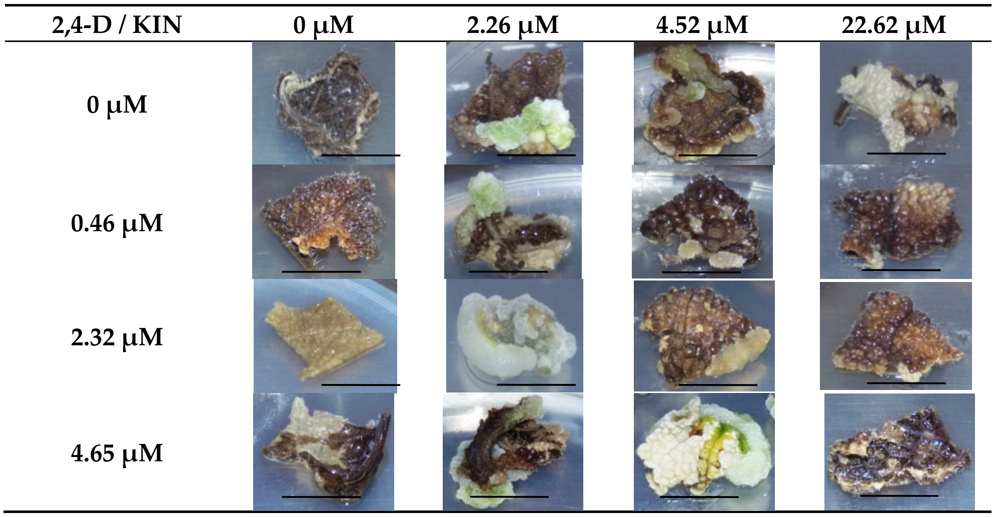

Figure S2. Callus of *S. tingitana* developed from leaf explants in light condition on MS supplemented with different combination of PGRs and 10 mg/L of ascorbic acid after 4 weeks. MS: Murashige and Skoog medium; PGRs: plant growth regulators; 2,4-D: 2,4-dichlorophenoxyacetic acid; KIN: kinetin. Bars = 1 cm.

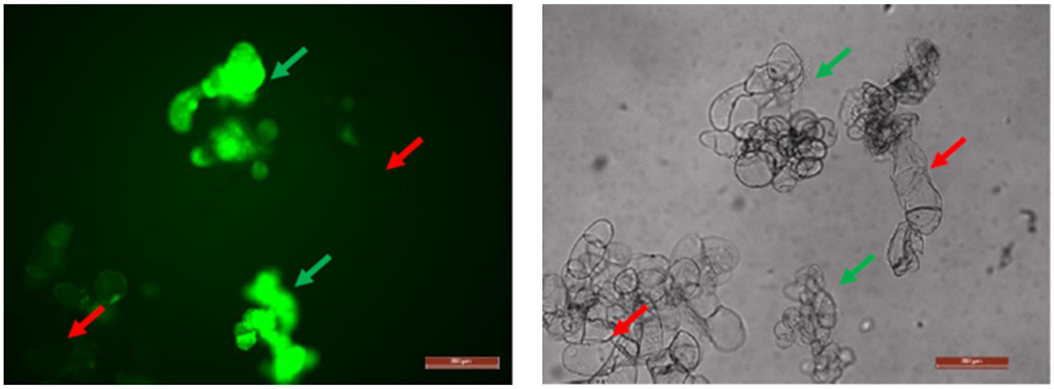

(**a**) (**b**)

Figure S3. FDA staining of *S. tingitana* callus.a: fluorescent observation; b: bright field observation (40×). Green arrows mean living cell while red mean dead cells. FDA: fluoresceine diacetate. Bars = 200 μm.

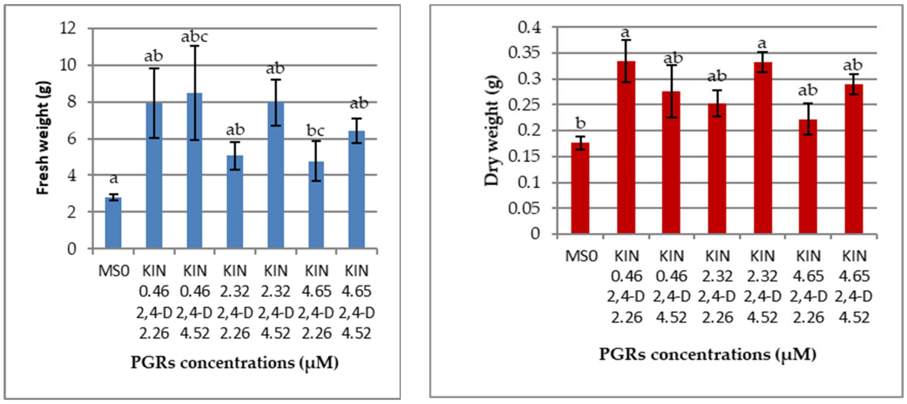

(**a**) (**b**)

Figure S4. Effect of different combination of plant growth regulators to *S. tingitana* callus biomass production.a: fresh weight; b: dry weight. Data are reported as mean of six replicates ± SE, n=6. Different letters identify values which differ at $p \leq 0.05$.

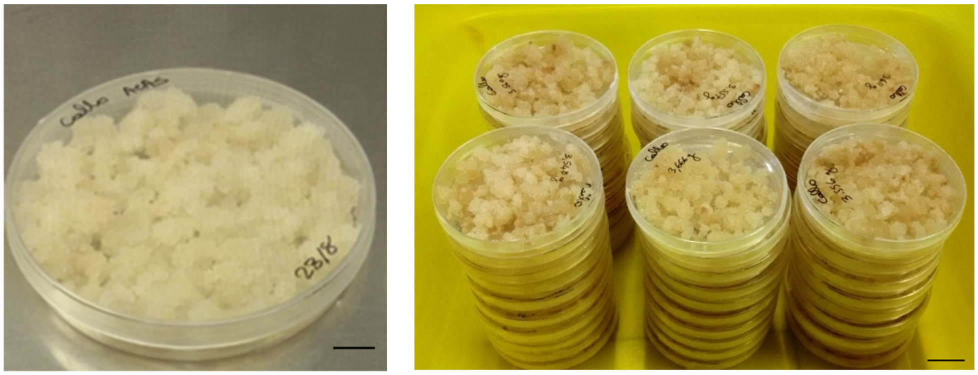

(**a**) (**b**)

Figure S5. Callus of *S. tingitana*. a: friable callus obtained after several subculture in MS medium supplemented with KIN 2.32 µM and 2,4-D 4.52 µM and ascorbic acid 10 mg/L. MS: Murashige and Skoog medium; KIN: kinetin; 2,4-D: 2,4-dichlorophenoxyacetic acid. Bar=1 cm. b: biomass production. Bar = 2 cm.

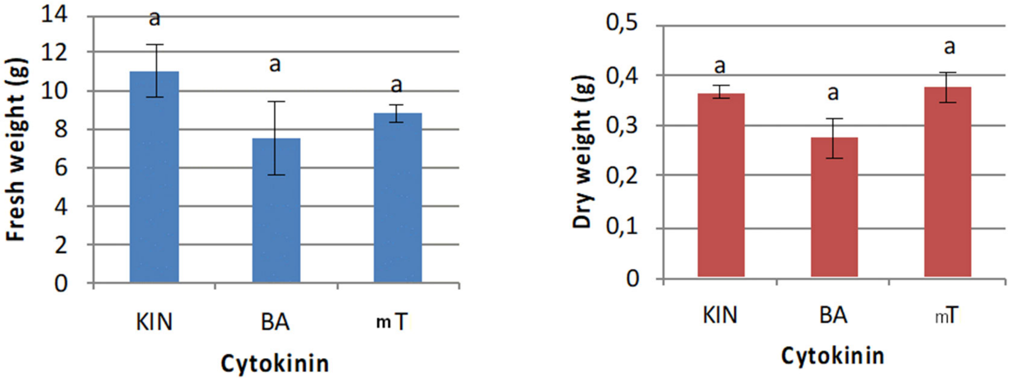

Figure S6. Effect of equimolar concentration of different cytokinins on callus growth. KIN: kinetin: BA: 6-benzylamino-purine; mT: meta Topolin. Values represent the mean ± standard error (SE) *n*=4.

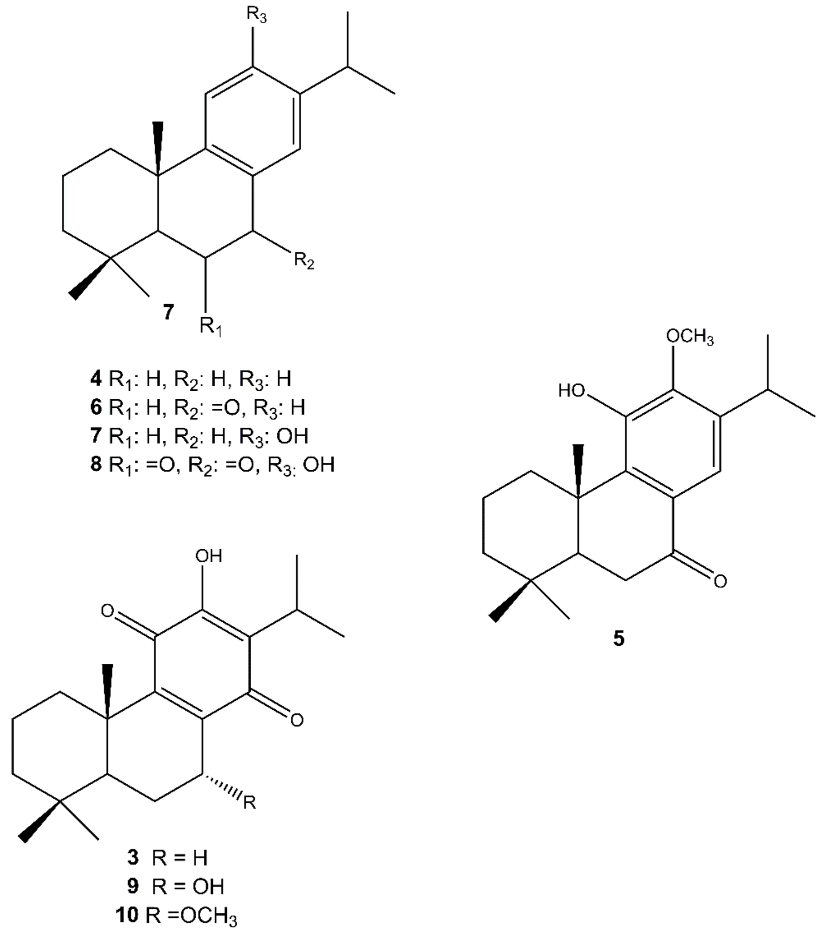

Figure S7. Abietane diterpenoids isolated from the roots of *S. tingitana*.**3**: royleanone; **4**: abieta-8,11,13-triene; **5**: cryptojaponol; **6**: abieta-8,11,13-trien-7-one; **7**: ferruginol; **8**: 12-hydroxyabieta-8,11,13-triene-6,7-dione (hypargenin C); **9**: horminone; **10**: 7-*O*-methylhorminone

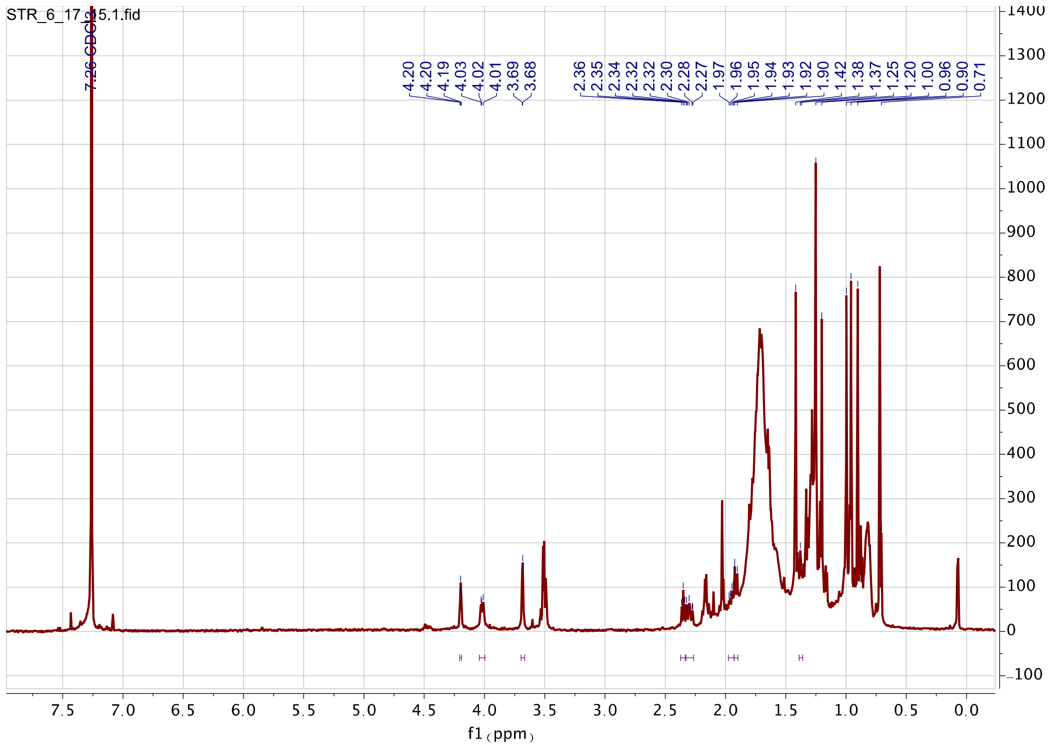

Figure S8. ^{1}H NMR (600 MHz, $CDCl_3$) spectrum of compound **13**.

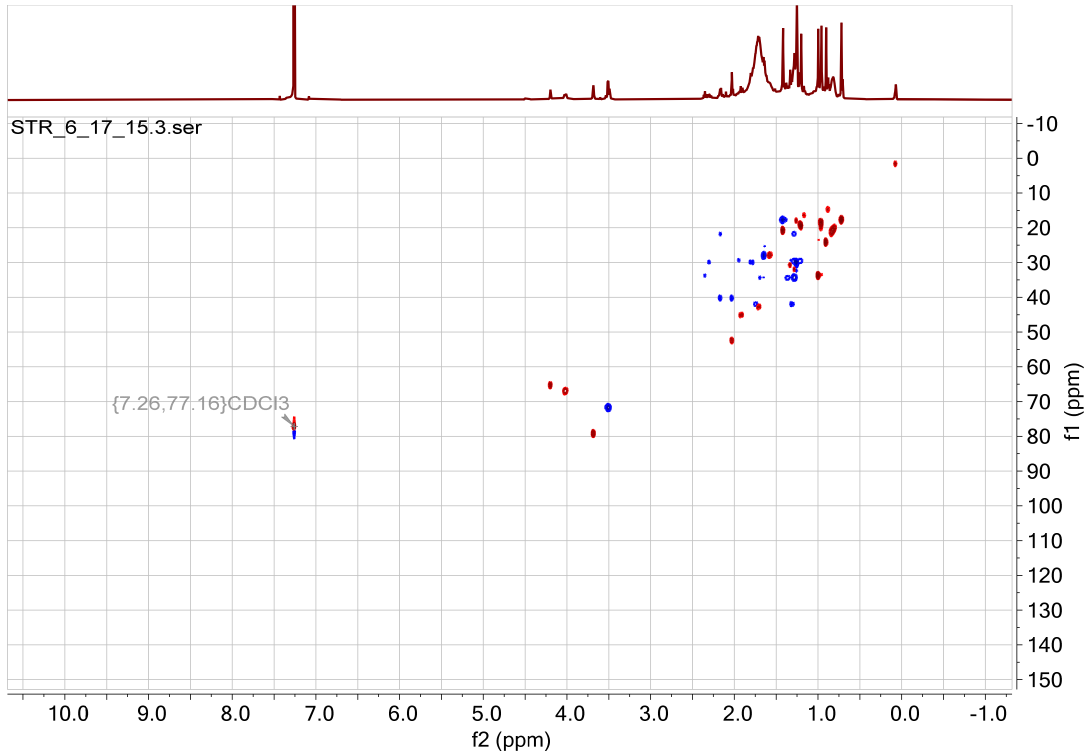

Figure S9. HSQC (600 MHz, $CDCl_3$) spectrum of compound **13**.

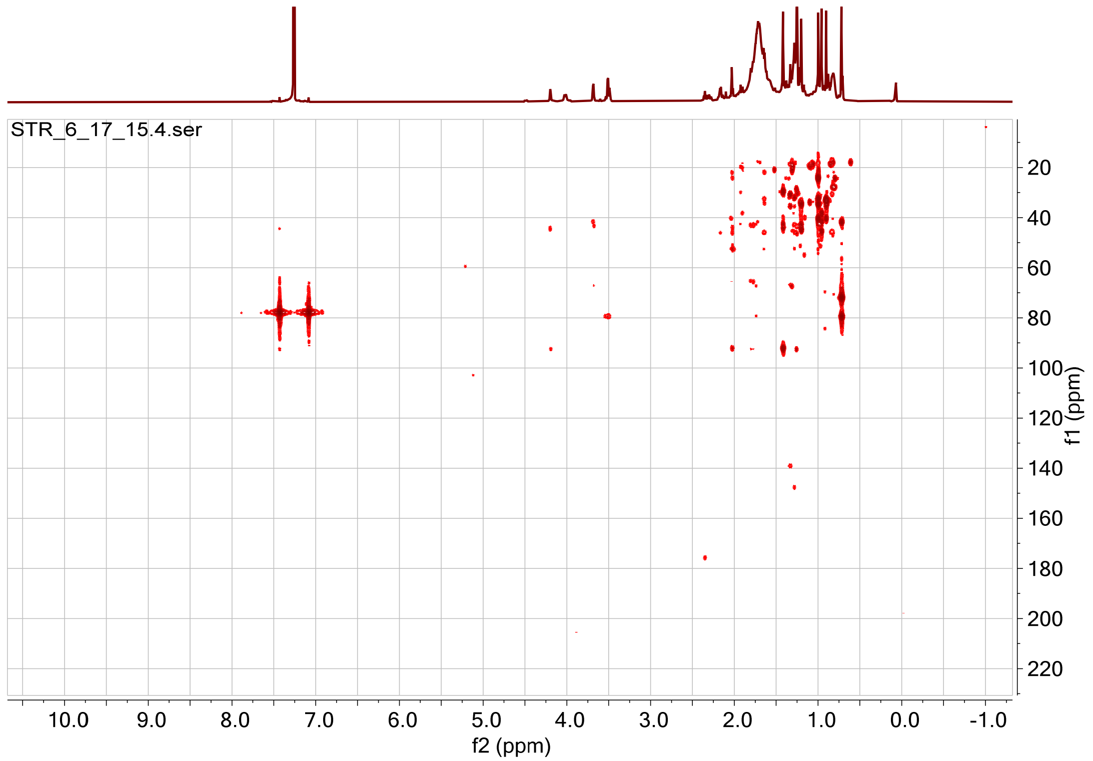

Figure S10. HMBC (600 MHz, $CDCl_3$) spectrum of compound **13**.

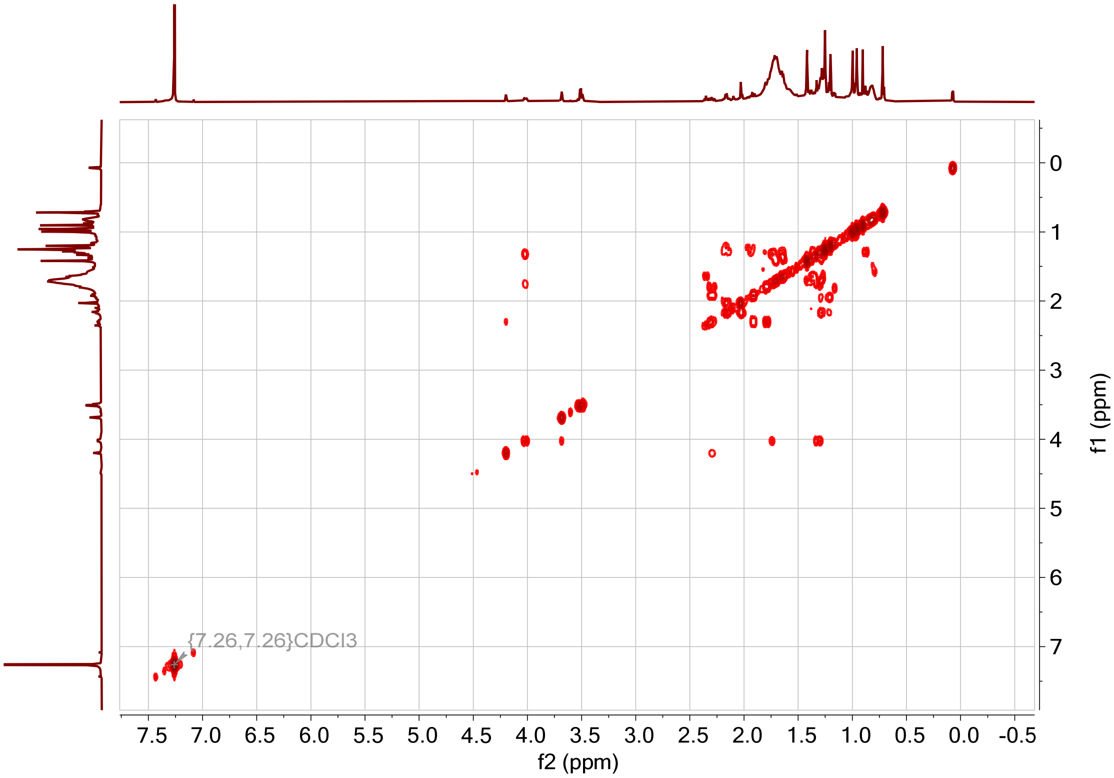

Figure S11. COSY (600 MHz, $CDCl_3$) spectrum of compound **13**.

.

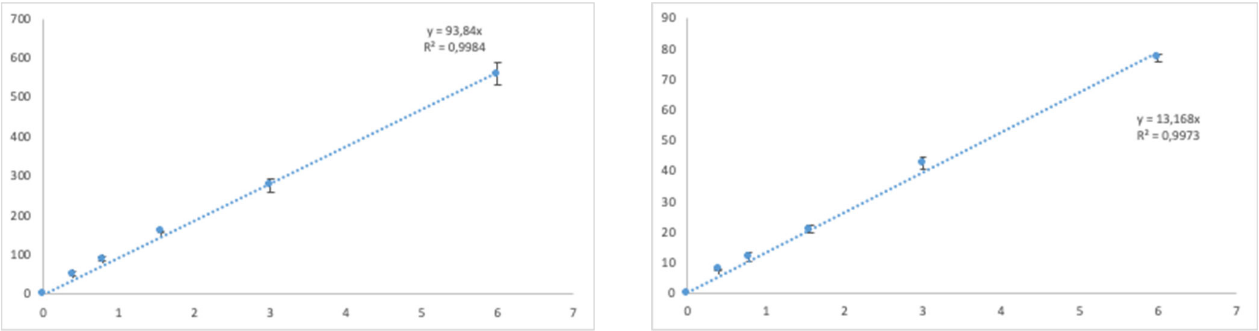

(1) (**b**)

Figure S12. Calibration curves obtained for the LC/MS/MS analysis of sclareol (**1**) and manool (**2**) using pure compounds.

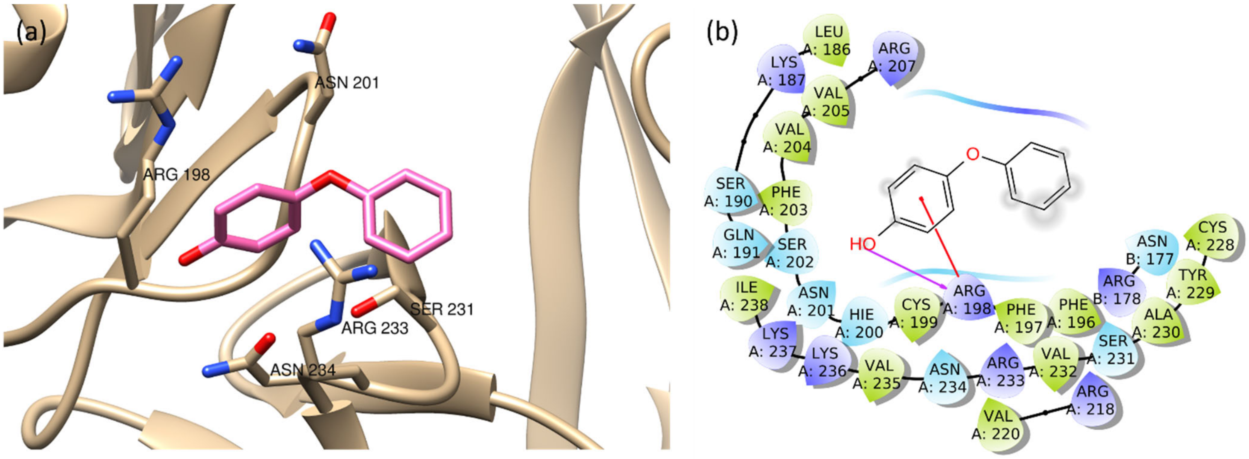

Figure S13. Binding pose and ligand interaction plot of 4-phenoxyphenol at the conserved AgrA active site.

(a): protein is reported as light brown ribbons, 4-phenoxyphenol is reported as capped sticks and is colored in magenta. (b): 4-phenoxyphenol is surrounded by the protein residues represented as follows: the negatively charged residues are indicated in red, polar residues are in cyan, hydrophobic residues are shown in green, H-bonds are presented as purple arrows and pi–cation as red line..

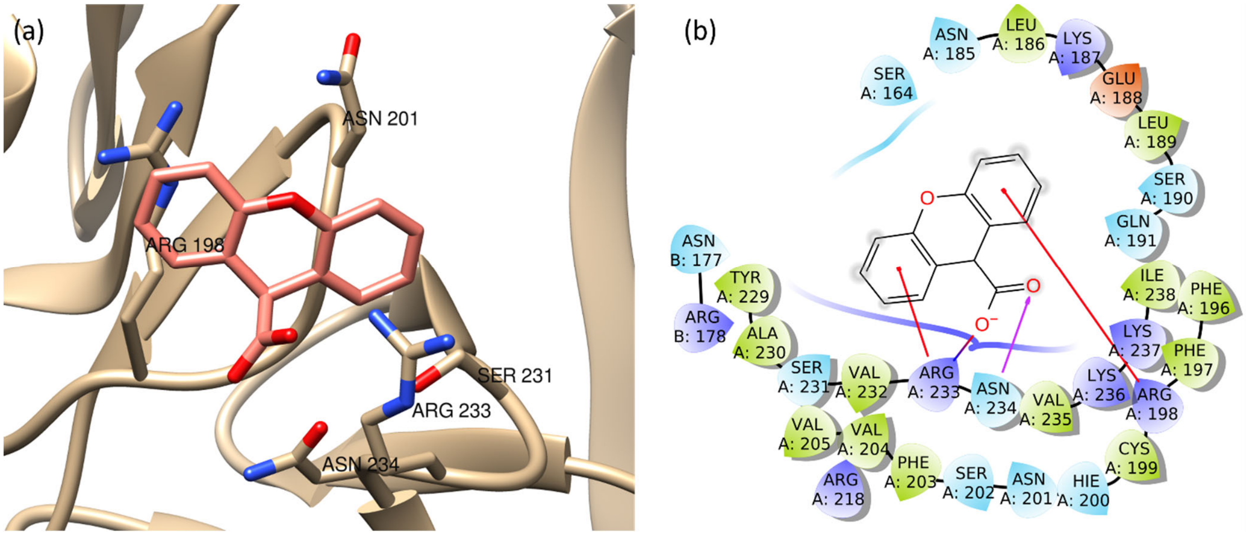

Figure S14. Binding pose and ligand interaction plot of 9H-xanthene-9-carboxylic acid at the conserved AgrA active site.(a): the protein is reported as light brown ribbons, 9H-xanthene-9-carboxylic acid is reported as capped sticks and is colored in salmon. (b): 9H-xanthene-9-carboxylic acid is surrounded by the residues represented as follows: the negatively charged residues are indicated in red, polar residues are in cyan, hydrophobic residues are shown in green, H-bonds are presented as purple arrows and pi–cation as red line.

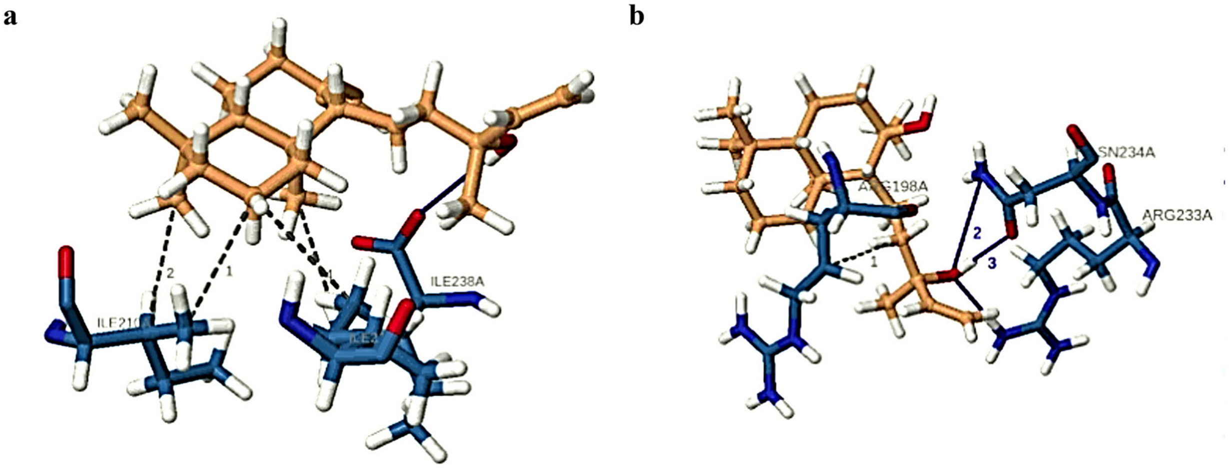

Figure S15. Manool and sclareol interactions with the key residues into the binding cavity.a: manool interactions; b: sclareol interactions; blue lines: H-bonds; broken grey lines: Van der Waals interactions.

Table S1. Literature survey about the presence of sclareol (**1**) and manool (**2**) in *Salvia* spp.[a]

Accepted Name, Protologue [1]	Synonyms [1]	Part/s of the Plant	Sclareol (1)	Manool (2)	References
***Salvia aethiopis* L.**, Sp. Pl.: 27 (1753)	Homotypic Names: *Sclarea aethiopis* (L.) Mill., Gard. Dict. ed. 8: n.º 2 (1768); *Sclarea lanata* Moench, Methodus: 374 (1794), nom. superfl.; *Salvia lanata* Stokes, Bot. Mat. Med.: 52 (1812), nom. illeg. Heterotypic Synonyms: *Salvia kochiana* Kunze, Index Seminum (LZ, Lipsiensis) 1847: 4 (1847); *Salvia leuconeura* Boiss., Diagn. Pl. Orient., ser. 2, 4: 20 (1859); *Aethiopis vera* Fourr., Ann. Soc. Linn. Lyon, n.s., 17: 134 (1869); *Salvia idanensis* Gand., Fl. Lyon.: 171 (1875).	flower, leaf and stems	ND	5.7% in stems, 0.6% p/V in flower, 2.1% in leaf of extract	[2]
		aerial parts	ND	0.8% of the essential oil	[3]
***Salvia argentea* L.**, Sp. Pl. ed. 2: 38 (1762)	Homotypic Names: *Sclarea argentea* (L.) Mill., Gard. Dict. ed. 8: n.º 15 (1768); *Salvia argentea* var. fontanesiana Maire in É.Jahandiez & al., Cat. Pl. Maroc 3: 642 (1934), not validly publ. Heterotypic Synonyms: *Salvia patula* Desf., Fl. Atlant. 1: 25 (1798); *Salvia atlantica* Pers., Syn. Pl. 1: 29 (1805); *Salvia tmolea* Boiss., Diagn. Pl. Orient. 5: 9 (1844); *Salvia saccata* Pourr. ex Willk. & Lange, Prodr. Fl. Hispan. 2: 424 (1868); *Salvia aurasiaca* Pomel, Nouv. Mat. Fl. Atl.: 306 (1874); *Salvia suaveolens* Pomel, Nouv. Mat. Fl. Atl.: 306 (1874); *Salvia argentea* var. *gussonei* Boiss. ex Nyman, Consp. Fl. Eur.: 569 (1881), nom. nud.; *Salvia argentea* var. *patula* (Desf.) Nyman, Consp. Fl. Eur.: 569 (1881); *Salvia alpestris* Hausskn. ex Nyman, Consp. Fl. Eur., Suppl. 2, 1: 248 (1889); *Salvia rhodopea* Velen., Sitzungsber. Königl. Böhm.	leaves and infiorescences	ND	14.6% of the essential oil	[4]

	Ges. Wiss., Math.-Naturwiss. Cl. 37: 388 (1892 publ. 1893); *Salvia argentea* subsp. *patula* (Desf.) Maire, Bull. Soc. Hist. Nat. Afrique N. 15: 90 (1924); *Salvia argentea* var. *pomelii* Maire, Bull. Soc. Hist. Nat. Afrique N. 15: 90 (1924); *Salvia argentea* var. *aurasiaca* (Pomel) Maire, Mém. Soc. Sci. Nat. Maroc 21-22: 13 (1930); *Salvia argentea* var. *mesatlantica* Maire, Mém. Soc. Sci. Nat. Maroc 21-22: 13 (1930).				
***Salvia atropatana* Bunge**, Labiat. Persic.: 47 (1873)	Heterotypic Synonyms: *Salvia bachtiarica* Bunge, Labiat. Persic.: 47 (1873); *Salvia hypochionea* Buhse ex Boiss., Fl. Orient. 4: 620 (1879); *Salvia montbretii* var. *virescens* Freyn, Bull. Herb. Boissier, sér. 2, 1: 278 (1901); *Salvia kopetdaghensis* Kudr., Trudy Sektora Rast. Res. Komit. Nauk Uzbeksk. SSR 3: 26 (1937); *Salvia linczevskii* Kudr., Trudy Sektora Rast. Res. Komit. Nauk Uzbeksk. SSR 3: 22 (1937); *Salvia kourossia* Parsa, Kew Bull. 3: 224 (1948); *Salvia lurorum* Rech.f., Oesterr. Bot. Z. 99: 57 (1952); *Salvia linguifolia* Hedge & Hub.-Mor., Notes Roy. Bot. Gard. Edinburgh 22: 181 (1957).	aerial parts	13.3% of the essential oil	1% of the essential oil	[5]
			0.3% of the essential oil	ND	[6]
Salvia candidissima* subsp. *candidissima	Heterotypic Synonyms: *Salvia albida* Jacq., Observ. Bot. 1: 10 (1764), name not found; *Salvia crassifolia* Sm. in J.Sibthorp & J.E.Smith, Fl. Graec. Prodr. 1: 17 (1806), nom. illeg.; *Salvia albida* Spreng., Index Seminum (HAL, Halensis) 1807: 53 (1807), nom. illeg.; *Salvia odorata* Willd., Enum. Pl.: 43 (1809); *Salvia argentea* Benth., Labiat. Gen. Spec.: 223 (1833), nom. illeg.; *Salvia armena* K.Koch, Linnaea 21: 654	roots	NQ	NQ	[7]

	(1849); *Salvia pycnophylla* Greuter & Burdet, Willdenowia 14: 301 (1984 publ. 1985).				
***Salvia candidissima* Vahl**, Enum. Pl. Obs. 1: 278 (1804)	Homotypic Names: *Sclarea candidissima* (Vahl) Soják, Cas. Nár. Mus., Odd. Prír. 152: 21 (1983).	aerial parts	2.5% of the essential oil	1.8% of the essential oil	[8]
***Salvia cassia* Sam. ex Rech.f.**, Ark. Bot., a.s., 1: 320 (1950)	-	aerial parts	ND	2.1% of the essential oil	[9]
***Salvia chrysophylla* Stapf**, Denkschr. Kaiserl. Akad. Wiss., Wien. Math.Naturwiss. Kl. 50: 96 (1885)	Heterotypic Synonyms: *Salvia bourgeana* Barbey, Bull. Soc. Vaud. Sci. Nat. 21: 96 (1885).	aerial parts	1.12% of the essential oil	1.37% of the essential oil	[10]
			NQ	ND	[11]
***Salvia desoleana* Atzei & V.Picci**, Webbia 36: 72 (1982)	-	aerial parts	ND	2.8% of the essential oil	[12]
***Salvia dominica* L.**, Sp. Pl.: 25 (1753)	Heterotypic Synonyms: *Salvia graveolens* Vahl, Enum. Pl. Obs. 1: 273 (1804). *Salvia commutata* Benth., Labiat. Gen. Spec.: 222 (1833). *Salvia syriaca* Gouan ex Benth. in A.P.de Candolle, Prodr. 12: 279 (1848).	whole plant	NQ	ND	[13]
***Salvia fruticosa* Mill.**, Gard. Dict. ed. 8: n.º 5 (1768)	Heterotypic Synonyms: *Salvia baccifera* Etl., Salv.: 18 (1777); *Salvia incarnata* Etl., Salv.: 25 (1777); *Salvia triloba* L.f., Suppl. Pl.: 88 (1782); *Salvia sipylea* Lam., Tabl. Encycl. 1: 68 (1791); *Salvia sypilea* Lam., Tabl. Encycl. 1: 68 (1791); *Salvia clusii* Jacq., Pl. Hort. Schoenbr. 2: 37 (1797); *Salvia marrubioides* Vahl, Enum. Pl. Obs. 1: 223 (1804); *Salvia ovata* F.Dietr., Nachtr. Vollst. Lex. Gärtn. 7: 465	whole plant	ND	NQ	[14]
		aerial parts	ND	0.2-4% of the essential oil	[15]
		leaves	ND	NQ	[16]

	(1821); *Salvia subtriloba* Schrank, Syll. Pl. Nov. 2: 58 (1826); *Sclarea triloba* (L.f.) Raf., Fl. Tellur. 3: 94 (1837); *Salvia libanotica* Boiss. & Gaill. in P.E.Boissier, Diagn. Pl. Orient., ser. 2, 4: 16 (1859); *Salvia cypria* Unger & Kotschy, Ins. Cypern: 266 (1865); *Salvia triloba* var. *calpeana* Dautez & Debeaux in J.O.Debeaux, Syn. Fl. Gibraltar: 161 (1889); *Salvia lobryana* Azn., Magyar Bot. Lapok 1: 195 (1902); *Salvia triloba* var. *subhastata* H.Lindb., Öfvers. Finska Vetensk.-Soc. Förh. 48(13): 94 (1906); *Salvia fruticosa* subsp. *cypria* (Unger & Kotschy) Holmboe, Stud. Veg. Cyprus: 158 (1914); *Salvia triloba* subsp. *cypria* (Kotschy) Holmboe, Stud. Veg. Cyprus: 158 (1914); *Salvia triloba* subsp. *libanotica* (Boiss. & Gaill.) Holmboe, Stud. Veg. Cyprus: 158 (1914); *Salvia thomasii* Lacaita, Nuovo Giorn. Bot. Ital., n.s., 29: 186 (1922 publ. 1923); *Salvia triloba* subsp. *calpeana* (Dautez & Debeaux) P.Silva, Agron. Lusit. 20: 237 (1958); *Salvia fruticosa* subsp. *thomasii* (Lacaita) Brullo, Guglielmo, Pavone & Terrasi, Inform. Bot. Ital. 26: 211 (1994 publ. 1995).				
***Salvia glutinosa* L.**, Sp. Pl.: 26 (1753)	Homotypic Names: *Sclarea glutinosa* (L.) Mill., Gard. Dict. ed. 8: n.º 11 (1768). *Glutinaria glutinosa* (L.) Raf., Fl. Tellur. 3: 93 (1837). *Drymosphace glutinosa* (L.) Opiz, Seznam: 38 (1852). Heterotypic Synonyms: *Glutinaria acuminata* Raf., Autik. Bot.: 122 (1840).	aerial parts	11.9% of the essential oil	6.8% of the essential oil	[8]
***Salvia hypoleuca* Benth.**,	-	roots	ND	NQ	[17]

A.P.de Candolle, Prodr. 12: 279 (1848)					
***Salvia judaica* Boiss.**, Diagn. Pl. Orient. 12: 61 (1853*)*	-	leaf	7.01% of the essential oil	ND	[18]
***Salvia lanigera* Poir.** J.B.A.M.de Lamarck, Encycl., Suppl. 5: 49 (1817)	Homotypic Names: *Salvia verbenaca* subsp. lanigera (Poir.) Batt. in J.A.Battandier & L.C.Trabut, Fl. Algérie, Dicot.: 688 (1890). Heterotypic Synonyms: *Salvia rugosissima* Zucc., Abh. Math.-Phys. Cl. Königl. Bayer. Akad. Wiss. 3: 244 (1843).	leaves	14.44% of ethanol extract	ND	[19]
***Salvia limbata* C.A.Mey.**, Verz. Pfl. Casp. Meer.: 86 (1831)	Heterotypic Synonyms: *Salvia flexuosa* Schrank, Syll. Pl. Nov. 2: 57 (1826), provisionally listed as a synonym; *Salvia polyadenia* Boiss. & Heldr. in P.E.Boissier, Diagn. Pl. Orient. 5: 7 (1844); *Salvia chrysadenia* Freyn, Bull. Herb. Boissier, sér. 2, 1: 279 (1901)	aerial parts	NQ	NQ	[20]
***Salvia majdae* (Rech.f. & Wendelbo) Sytsma**, Taxon 66: 142 (2017)		roots	ND	NQ	[21]
			ND	NQ	[22]
	Homotypic Names: *Zhumeria majdae* Rech.f. & Wendelbo, Nytt Mag. Bot. 14: 39 (1967).	aerial parts	ND	37.1% of essential oil	[23]
***Salvia macrosiphon* Boiss.**, Diagn. Pl. Orient. 5: 11 (1844)	Heterotypic Synonyms: *Salvia kotschyi* Boiss., Diagn. Pl. Orient. 7: 46 (1846); *Salvia macrosiphon* var. *cabulica* Benth. in A.P.de Candolle, Prodr. 12: 282 (1848); *Salvia macrosiphon* var. *kotschyi* (Boiss.) Boiss., Fl. Orient. 4: 615 (1879); *Salvia macrosiphonia* St.-Lag., Ann. Soc. Bot. Lyon 7: 134 (1880); *Salvia cuspidatissima* Pau, Trab. Mus. Ci. Nat., Ser. Bot. 14: 33 (1918); *Salvia albifrons* Nábelek, Spisy Prír. Fak. Masarykovy Univ. 70: 49 (1926); *Salvia macrosiphon* var. *brachycalycina* Bornm., Bot. Jahrb.	aerial parts	15.76 % of essential oil	2.10% of essential oil	[24]
			8.6% % of essential oil	ND	[6]

	Syst. 62: 238 (1934); *Salvia macrosiphon* var. *glandulosissima* Bornm., Bot. Jahrb. Syst. 62: 238 (1934); *Salvia nachiczevanica* Pobed. in V.L.Komarov, Fl. URSS 21: 657 (1954)				
***Salvia montbretii* Benth.**, Ann. Sci. Nat., Bot., sér. 2, 6: 42 (1836)	-	aerial parts	ND	0.3 % of essential oil	[25]
***Salvia multicaulis* Vahl,** Enum. Pl. Obs. 1: 225 (1804)	Homotypic Names: *Arischrada multicaulis* (Vahl) Pobed., Novosti Sist. Vyssh. Rast. 9: 247 (1972); *Stiefia multicaulis* (Vahl) Soják, Cas. Nár. Mus., Odd. Prír. 152: 22 (1983). Heterotypic Synonyms: *Salvia pinardii* Boiss., Diagn. Pl. Orient. 12: 59 (1853); *Salvia rascheyana* Boiss., Diagn. Pl. Orient. 12: 58 (1853); *Salvia bodeana* Bunge, Labiat. Persic.: 42 (1873); *Salvia szovitsiana* Bunge, Labiat. Persic.: 43 (1873); *Schraderia acetabulosa* Pobed. in V.L.Komarov, Fl. URSS 21: 369 (1954).	roots	ND	NQ	[26]
		stems and leaves	ND	NQ	[27]
***Salvia officinalis* L.**, Sp. Pl.: 23 (1753)	Heterotypic Synonyms: *Salvia officinalis* subsp. minor (C.C.Gmel.) Gams in G.Hegi, Ill. Fl. Mitt.-Eur. 5: 2483 (1927).	leaf	5.18% of essential oil *S. officinalis*, 2.86% *S. o*. cv. 'Purpurascens', 3.08% *S. o*. cv. 'Tricolor', 2.16% *S. o*. cv. 'Kew Gold', 7.01 % *S. judaica*	ND	[18]
		aerial parts	NQ	NQ	[28]
		leaves	ND	NQ	[29]
		flower, leaf and stem	ND	9.0% (leaf), 11.1 % (flower), 9.9%	[30]

				(stem) of ethanol extract				
***Salvia oligophylla* Aucher ex Benth.**, A.P.de Candolle, Prodr. 12: 279 (1848)	Heterotypic Synonyms: *Salvia sinaica* Delile ex Benth., Labiat. Gen. Spec.: 718 (1835); *Salvia lorentii* Hochst. in J.A.Lorent, Wanderungen: 333 (1845); *Salvia sieberi* C.Presl, Abh. Königl. Böhm. Ges. Wiss., ser. 5, 3: 530 (1845); *Salvia rassamii* Boiss., Fl. Orient. 4: 615 (1879); *Salvia alliaria* Parsa, Kew Bull. 3: 224 (1948).	aerial parts	ND	3.99% of essential oil	[6]	Aerial parts	ND	Non spe[illegible] ficata[illegible]
***Salvia palaestina* Benth.**, Labiat. Gen. Spec.: 718 (1835)	Heterotypic Synonyms: *Salvia sinaica* Delile ex Benth., Labiat. Gen. Spec.: 718 (1835); *Salvia lorentii* Hochst. in J.A.Lorent, Wanderungen: 333 (1845); *Salvia sieberi* C.Presl, Abh. Königl. Böhm. Ges. Wiss., ser. 5, 3: 530 (1845); *Salvia rassamii* Boiss., Fl. Orient. 4: 615 (1879); *Salvia alliaria* Parsa, Kew Bull. 3: 224 (1948).	leaves	NQ	ND	[31]	Aerial parts	ND	Non spe[illegible] ficata[illegible]
***Salvia patens* Cav.**, Icon. 5: 33 (1799)	Heterotypic Synonyms: *Salvia grandiflora* Née ex Cav., Icon. 5: 33 (1799), nom. illeg. *Salvia spectabilis* Kunth in F.W.H.von Humboldt, A.J.A.Bonpland & C.S.Kunth, Nov. Gen. Sp. 2: 304 (1818). *Salvia macrantha* Schltdl., Allg. Gartenzeitung 6: 314 (1838). *Salvia decipiens* M.Martens & Galeotti, Bull. Acad. Roy. Sci. Bruxelles 11(2): 64 (1844). *Salvia staminea* M.Martens & Galeotti, Bull. Acad. Roy. Sci. Bruxelles 11(2): 65 (1844), nom. illeg. *Salvia mendax* Epling, Repert. Spec. Nov. Regni Veg. Beih. 110: 96 (1938).	whole plant	4.85% of essential oil	ND	[32]			
***Salvia persepolitana* Boiss.**, Diagn. Pl. Orient. 12: 60 (1853)	-	aerial parts	0.5% of essential oil	37.3% of essential oil	[33]			

***Salvia poculata* Nábelek**, Spisy Prír. Fak. Masarykovy Univ. 70: 50 (1926)	Heterotypic Synonyms: *Salvia brevidens* Hedge & Hub.-Mor., Notes Roy. Bot. Gard. Edinburgh 22: 183 (1957).	aerial parts	NQ	ND	[34]
***Salvia reuteriana* Boiss.**, Diagn. Pl. Orient. 5: 10 (1844)	-	aerial parts	0.77 - 7.88% of essential oil	ND	[35]
			2% of essential oil	ND	[36]
			NQ	ND	[37]
***Salvia runcinata* L.f.**, Suppl. Pl.: 89 (1782)	Heterotypic Synonyms: *Salvia monticola* Benth. in E.H.F.Meyer, Comm. Pl. Afr. Austr.: 238 (1838). *Salvia runcinata* var. *major* Benth. in A.P.de Candolle, Prodr. 12: 352 (1848). *Salvia runcinata* var. *grandiflora* Skan in W.H.Harvey & auct. suc. (eds.), Fl. Cap. 5(1): 327 (1910). *Salvia runcinata* var. *nana* Skan in W.H.Harvey & auct. suc. (eds.), Fl. Cap. 5(1): 327 (1910). *Salvia sisymbrifolia* Skan in W.H.Harvey & auct. suc. (eds.), Fl. Cap. 5(1): 328 (1910).	aerial parts	ND	6.4% of essential oil	[38]
***Salvia sahendica* Boiss. & Buhse**, Nouv. Mém. Soc. Imp. Naturalistes Moscou 12: 172 (1860)	-	aerial parts	NQ	ND	[39]
			NQ	ND	[40]
***Salvia santolinifolia* Boiss.**, Diagn. Pl. Orient. 5: 13 (1844)	Homotypic Names: *Pleudia santolinifolia* (Boiss.) M.Will, N.Schmalz & Class.-Bockh., Turkish J. Bot. 39: 703 (2015).	aerial parts	2.75% of essential oil	ND	[41]
***Salvia sclarea* L.**, Sp. Pl.: 27 (1753)	Homotypic Names: *Sclarea vulgaris* Mill., Gard. Dict. ed.8: nº1 (1768); *Aethiopis sclarea* (L.) Opiz, Seznam: 11 (1852). Heterotypic Names: *Salvia haematodes* Scop., Fl. Carniol., ed.2, 1: 29 (1771),	whole plant	NQ	NQ	[42]
			ND	NQ	[43]
		aerial parts	5.3% of essential oil	ND	[6]

	nom. illeg.; *Salvia coarctata* Vahl, Enum. Pl. Obs. 1: 253 (1804); *Salvia simsiana* Schult., Mant. 1: 210 (1822); *Salvia calostachya* Gand., Fl. Lyon.: 171 (1875); *Salvia sclarea* var. *calostachya* (Gand.) Nyman, Consp. Fl. Eur.: 569 (1881); *Salvia turkestanica* Noter, Rev. Hort. (Paris) 77: 502 (1905); *Salvia sclarea* var. *turkestaniana* Mottet, Rev. Hort. (Paris) 79: 135 (1907); *Salvia lucana* Cavara & Grande, Bull. Orto Bot. Regia Univ. Napoli 3: 436 (1913); *Salvia pamirica* Gand., Bull. Soc. Bot. France 60: 26 (1913); *Salvia altilabrosa* Pan, Trab. Mus. Ci. Nat., Ser. Bot. 14: 33 (1918).				
***Salvia sclareoides* Brot.**, Fl. Lusit. 1: 17 (1804)	Homotypic Names: *Salvia pratensis* var. sclareoides (Brot.) Briq., Lab. Alp. Mar.: 532 (1895). Heterotypic Synonyms: *Sclarea lusitanica* Mill., Gard. Dict. ed. 8: n.º 3 (1768). *Salvia bullata* Vahl, Enum. Pl. Obs. 1: 265 (1804), nom. illeg. *Salvia lusitanica* Poir. in J.B.A.M.de Lamarck, Encycl. 6: 606 (1805). *Salvia elongata* Spreng., Pl. Min. Cogn. Pug. 1: 3 (1813), nom. illeg. *Salvia lusitanica* J.Jacq., Ecl. Pl. Rar. 1: 47 (1813), sensu auct. *Salvia baetica* Boiss., Elench. Pl. Nov.: 73 (1838). *Salvia pratensis* var. *bullata* Briq., Lab. Alp. Mar.: 531 (1895). *Salvia pratensis* var. *lusitanica* Briq., Lab. Alp. Mar.: 532 (1895). *Salvia sclareoides* var. *baetica* (Boiss.) Figuerola, Collect. Bot. (Barcelona) 17: 309 (1988 publ. 1989).	aerial parts	0.3% of essential oil	ND	[44]
***Salvia sharifii* Rech.f. & Esfand.**, Oesterr. Bot. Z. 99: 55 (1952)	-	seeds	21.9% of essential oil	ND	[45]

***Salvia staminea* Montbret & Aucher ex Benth.**, Ann. Sci. Nat., Bot., sér. 2, 6: 41 (1836)	Heterotypic Synonyms: *Salvia calvertii* Boiss., Fl. Orient. 4: 626 (1879). *Salvia staminea* subsp. *armeniaca* Bordz., Trudy Bot. Sada Imp. Yur'evsk. Univ. 13: 22 (1912). *Salvia armeniaca* (Bordz.) Grossh., Beih. Bot. Centralbl. 44(2): 237 (1927). *Salvia kudjurica* Rech.f., Oesterr. Bot. Z. 99: 56 (1952). *Salvia transcaucasica* Pobed., Bot. Mater. Gerb. Bot. Inst. Komarova Akad. Nauk S.S.S.R. 21: 321 (1961).	aerial parts	da 0.01 a 3.1% of essential oil	ND	[46]
***Salvia stenophylla* Burch. ex Benth., Labiat. Gen. Spec.: 306 (1833)**	Heterotypic Synonyms: *Salvia chlorophylla* Briq., Bull. Herb. Boissier, sér. 2, 3: 1080 (1903). *Salvia xerobia* Briq., Bull. Herb. Boissier, sér. 2, 3: 1076 (1903). *Salvia stenophylla* var. *subintegra* Skan in W.H.Harvey & auct. suc. (eds.), Fl. Cap. 5(1): 326 (1910). *Salvia pallida* Dinter, Repert. Spec. Nov. Regni Veg. 23: 227 (1926), nom. nud.	aerial parts	ND	3.2 -12.7% of essential oil	[38]
***Salvia verticillata* L., Sp. Pl.: 26 (1753)**	Homotypic Names: *Horminum verticillatum* (L.) Mill., Gard. Dict. ed. 8: n.º 3 (1768); *Covola verticillata* (L.) Medik., Philos. Bot. 2: 67 (1791); *Hemisphace verticillata* (L.) Opiz, Seznam: 50 (1852); *Sphacopsis verticillata* (L.) Briq., Lab. Alp. Mar.: 184 (1891)	roots	ND	NQ	[47]

[a] ND: not detected; NQ: isolated, but quantification non reported.

References

1. Govaerts, R. World Checklist of Selected Plant Families. **2019**.
2. Veličković, D.; Ranđelović , N.; Ristić, M.; Šmelcerović, A.; Veličković, A.S. Chemical composition and antimicrobial action of the ethanol extracts of *Salvia pratensis* L., *Salvia glutinosa* L. and *Salvia aethiopis* L. *J. Serb. Chem. Soc.* **2002**, *67*, 639-646.
3. Morteza-Semnani, K.; Moshiri, K.; Akbarzadeh, M. The Essential Oil Composition of *Salvia multicaulis* Vahl. *J. Essent. Oil Bear. Pl.* **2005**, *8*, 6-10, doi:10.1080/0972060X.2005.10643412.
4. Couladis, M.; Tzakou, O.; Stojanovic, D.; Mimica-Dukic, N.; Jancic, R. The essential oil composition of *Salvia argentea* L. *Flavour Fragrance J.* **2001**, *16*, 227-229, doi:https://doi.org/10.1002/ffj.989.
5. Mirza, M.; Ahmadi, L. Composition of the Essential Oil of *Salvia atropatana* Bunge. *J. Essent. Oil Res.* **2000**, *12*, 575-576, doi:10.1080/10412905.2000.9712162.
6. Salimpour, F.; Mazooji, A.; Akhoondi, S. Chemotaxonomy of six *Salvia* species using essential oil composition markers. *J. Med. Plant. Res.* **2011**, *5*, 1795-1805.
7. Ulubelen, A.; Tan, N.; Topcu, G. Terpenoids from *Salvia candidissima* subsp. *candidissima*. *Phytochemistry* **1997**, *45*, 1221-1223.
8. Pitarokili, D.; Tzakou, O.; Loukis, A. Essential oil composition of *Salvia verticillata*, *S. verbenaca*, *S. glutinosa* and *S. candidissima* growing wild in Greece. *Flavour Fragrance J.* **2006**, *21*, 670-673, doi:https://doi.org/10.1002/ffj.1647.
9. Şen Utsukarci, B.; Gurda, B.; Bilgin, M.; Satana, D.; Demirci, B.; Tan, N.; Mat, A. Biological Activities of Various Extracts from *Salvia cassia* Sam. ex Rech.f. and Chemical Composition of Its Most Active Extract. *Rec. Nat. Prod.* **2019**, *13*, 24-36, doi:10.25135/rnp.68.18.02.090.
10. Duru, M.; Tel Çayan, G.; Öztürk, M.; Harmandar, M. Chemical Composition, Antioxidant and Anticholinesterase Activities of the Essential Oil of *Salvia chrysophylla* Staph. *Rec. Nat. Prod.* **2012**, *6*, 175.
11. Çulhaoğlu, B.; Yapar, G.; Dirmenci, T.; Topçu, G. Bioactive constituents of *Salvia chrysophylla* Stapf. *Nat. Prod. Res.* **2013**, *27*, 438-447, doi:10.1080/14786419.2012.734820.
12. Marongiu, B.; Porcedda, S.; Porta, G.D.; Reverchon, E. Extraction and isolation of *Salvia desoleana* and *Mentha spicata* subsp. insularis essential oils by supercritical CO2. *Flavour Fragrance J.* **2001**, *16*, 384-388, doi:https://doi.org/10.1002/ffj.1021.
13. Hasan, M.; Al-Jaber, H.; Al-Qudah, M.; Zarga, M. New sesterterpenoids and other constituents from *Salvia dominica* growing wild in Jordan. *Phytochem. Lett.* **2016**, *16*, 12-17, doi:10.1016/j.phytol.2016.02.009.
14. Topçu, G.; Öztürk, M.; Kuşman, T. Terpenoids, essential oil composition, fatty acid profile, and biological activities of Anatolian *Salvia fruticosa* Mill. *Turk. J. Chem.* **2013**, *37*, 619-632.
15. Pitarokili, D.; Tzakou, O.; Loukis, A.; Harvala, C. Volatile metabolites from *Salvia fruticosa* as antifungal agents in soilborne pathogens. *Journal of Agricultural and Food Chemistry* **2003**, *51*, 3294-3301, doi:10.1021/jf0211534.
16. Abou-Jawdah, Y.; Sobh, H.; Salameh, A. Antimycotic activities of selected plant flora, growing wild in Lebanon, against phytopathogenic fungi. *Journal of Agricultural and Food Chemistry* **2002**, *50*, 3208-3213, doi:10.1021/jf0115490.
17. Saeidnia, S.; Ghamarinia, M.; Gohari, A.R.; Shakeri, A. Terpenes From the Root of *Salvia hypoleuca* Benth. *Daru* **2012**, *20*, 66, doi:10.1186/2008-2231-20-66.
18. Böszörményi, A.; Héthelyi, E.; Farkas, A.; Horváth, G.; Papp, N.; Lemberkovics, E.; Szoke, E. Chemical and genetic relationships among sage (*Salvia officinalis* L.) cultivars and Judean sage (*Salvia judaica* Boiss.). *Journal of Agricultural and Food Chemistry* **2009**, *57*, 4663-4667, doi:10.1021/jf9005092.
19. Alonazi, M.A.; Jemel, I.; Moubayed, N.; Alwhibi, M.; El-Sayed, N.N.E.; Ben Bacha, A. Evaluation of the in vitro anti-inflammatory and cytotoxic potential of ethanolic and aqueous extracts of *Origanum syriacum* and Salvia *lanigera* leaves. *Environ. Sci. Pollut. Res. Int.* **2021**, *28*, 19890-19900, doi:10.1007/s11356-020-11961-z.
20. Ulubelen, A.; Topcu, G.; Sonmez, U.; Eris, C.; Ozgen, U. Norsesterterpenes and Diterpenes from the Aerial Parts of *Salvia limbata*. *Phytochemistry* **1996**, *43*, 431-434.
21. Rustaiyan, A.; Samadizadeh, M.; Habibi, Z.; Jakupovic, J. Two diterpenes with rearranged abietane skeletons from *Zhumeria majdae*. *Phytochemistry* **1995**, *39*, 163-165, doi:https://doi.org/10.1016/0031-9422(94)00692-M.
22. Zadali, R.; Nejad-Ebrahimi, S.; Hadjiakhoondi, A.; Fiengo, L.; D'Ambola, M.; De Vita, S.; Tofighi, Z.; Chini, M.G.; Bifulco, G.; De Tommasi, N. Diterpenoids from *Zhumeria majdae* roots as potential heat shock protein 90 (HSP90) modulators. *Phytochemistry* **2021**, *185*, 112685, doi:https://doi.org/10.1016/j.phytochem.2021.112685.
23. Javidnia, K.; Moein, M.R.; Ayatollahi, M.; Moein, S.; Amoozegar, Z.; Rustaiyan, A. Constituents of Stem Oil of *Zhumeria majdae* Rech. from Iran. *J. Essent. Oil Res.* **2006**, *18*, 91-92, doi:10.1080/10412905.2006.9699395.
24. Sefidkon, F.; Mirza, M.; Javidtash, I. Essential oil Composition of *Salvia macrosiphon* Boiss. from Iran. *J. Essent. Oil Bear. Pl.* **2005**, *8*, 126-129, doi:10.1080/0972060X.2005.10643431.
25. Abak, F.; Yildiz, G.; Atamov, V.; Kurkcuoglu, M. Composition of the Essential Oil of *Salvia montbretii* Benth. from Turkey. *Rec. Nat. Prod.* **2018**, *12*, 426-431, doi:http://dx.doi.org/10.25135/rnp.51.17.12.080.
26. Ulubelen, A.; Topcu, G.; Johansson, C.B. Norditerpenoids and diterpenoids from *Salvia multicaulis* with antituberculous activity. *Journal of Natural Products* **1997**, *60*, 1275-1280, doi:10.1021/np9700681.
27. Christensen, K.B.; Jørgensen, M.; Kotowska, D.; Petersen, R.K.; Kristiansen, K.; Christensen, L.P. Activation of the nuclear receptor PPARγ by metabolites isolated from sage (*Salvia officinalis* L.). *J. Ethnopharmacol.* **2010**, *132*, 127-133, doi:10.1016/j.jep.2010.07.054.

28. Moreira, M.R.; Souza, A.B.; Moreira, M.A.; Bianchi, T.C.; Carneiro, L.J.; Estrela, F.T.; dos Santos, R.A.; Januário, A.H.; Martins, C.H.G.; Ambrosio, S.R.; et al. RP-HPLC analysis of manool-rich *Salvia officinalis* extract and its antimicrobial activity against bacteria associated with dental caries. *Rev. Bras. Farma.* **2013**, *23*, 870-876, doi:https://doi.org/10.1590/S0102-695X2013000600003.
29. Nicolella, H.D.; de Oliveira, P.F.; Munari, C.C.; Costa, G.F.; Moreira, M.R.; Veneziani, R.C.; Tavares, D.C. Differential effect of manool -a diterpene from *Salvia officinalis*, on genotoxicity induced by methyl methanesulfonate in V79 and HepG2 cells. *Food Chem. Toxicol.* **2014**, *72*, 8-12, doi:10.1016/j.fct.2014.06.025.
30. Veličković, T.; Randjelović, V.; Ristić, S.; Veličković, S.; Šmelcerović, A. Chemical constituents and antimicrobial activity of the ethanol extracts obtained from the flower, leaf and stem of Salvia officinalis L. *J. Serb. Chem. Soc.* **2003**, *68*, 17-24.
31. Ulubelen, A. Terpenoids from *Salvia palaestina*. *Phytochemistry* **1985**, *24*, 1386-1387.
32. Ulaş Çolak, N.; Yıldırım, S.; Bozdeveci, A.; Yaylı, N.; Coskuncelebi, K.; Fandaklı, S.; Yaşar, A. Essential Oil Composition, Antimicrobial and Antioxidant Activities of *Salvia staminea*. *Rec. Nat. Prod.* **2017**, *12*, 86-94, doi:10.25135/rnp.08.17.03.013.
33. Habibi, Z.; Yousefi, M.; Aghaie, H.R.; Salehi, P.; Masoudi, S.; Rustaiyan, A. Chemical Composition of Essential Oil of *Salvia persepolitana* Boiss. and *Salvia rhytidea* Benth. from Iran. *J. Essent. Oil Res.* **2008**, *20*, 1-3, doi:10.1080/10412905.2008.9699405.
34. Kolak, U.; Hac1bekirp lu, I.; Öztürk, M.; Özgökçe, F.; Topçu, G.; Ulubelen, A. Antioxidant and anticholinesterase constituents of *Salvia poculata*. *Turk. J. Chem.* **2009**, *33*, 813-823.
35. Fattahi, B.; Nazeri, V.; Kalantari, S.; Bonfill, M.; Fattahi, M. Essential oil variation in wild-growing populations of *Salvia reuterana* Boiss. collected from Iran: Using GC–MS and multivariate analysis. *Ind. Crop. Prod.* **2016**, *81*, 180-190, doi:https://doi.org/10.1016/j.indcrop.2015.11.061.
36. Karamian, R.; Asadbegy, M.; Pakzad, R.; Ranjbar, M. Essential oil compositions and in vitro antioxidant and antibacterial activities of the methanol extracts of two *Salvia* species (Lamiaceae) from Iran. *International Journal of Agriculture and Crop Sciences (IJACS)* **2013**, *5*.
37. Farimani, M.M.; Miran, M. Labdane diterpenoids from *Salvia reuterana*. *Phytochemistry* **2014**, *108*, 264-269, doi:10.1016/j.phytochem.2014.08.024.
38. Viljoen, A.M.; Gono-Bwalya, A.; Kamatou, G.P.P.; Başer, K.H.C.; Demirci, B. The Essential Oil Composition and Chemotaxonomy of *Salvia stenophylla* and its Allies *S. repens* and *S. runcinata*. *J. Essent. Oil Res.* **2006**, *18*, 37-45, doi:10.1080/10412905.2006.12067117.
39. Moghaddam, F.M.; Zaynizadeh, B.; Ruedi, P. Salvileucolide methylester, a sesterterpene from *Salvia Sahendica*. *Phytochemistry* **1995**, *39*, 715-716.
40. Mofidi Tabatabaei, S.; Salehi, P.; Moridi Farimani, M.; Neuburger, M.; De Mieri, M.; Hamburger, M.; Nejad-Ebrahimi, S. A nor-diterpene from *Salvia sahendica* leaves. *Nat. Prod. Res.* **2017**, *31*, 1758-1765, doi:10.1080/14786419.2017.1290619.
41. Nadir, M.; Rasheed, M.; Sherwani, S.K.; Kazmi, S.U.; Ahmad, V.U. Chemical and antimicrobial studies on the essential oil from *Salvia santolinifolia* Boiss. *Pak. J. Pharm. Sci.* **2013**, *26*, 39-52.
42. Ulubelen, A.; Topcu, G.; Eriş, C.; Sönmez, U.; Kartal, M.; Kurucu, S.; Bozok-Johansson, C. Terpenoids from *Salvia sclarea*. *Phytochemistry* **1994**, *36*, 971-974, doi:10.1016/s0031-9422(00)90474-6.
43. Noori, S.; Hassan, Z.M.; Mohammadi, M.; Habibi, Z.; Sohrabi, N.; Bayanolhagh, S. Sclareol modulates the Treg intra-tumoral infiltrated cell and inhibits tumor growth in vivo. *Cell. Immunol.* **2010**, *263*, 148-153, doi:10.1016/j.cellimm.2010.02.009.
44. Sepahvand, R.; Delfan, B.; Ghanbarzadeh, S.; Rashidipour, M.; Veiskarami, G.H.; Ghasemian-Yadegari, J. Chemical composition, antioxidant activity and antibacterial effect of essential oil of the aerial parts of *Salvia sclareoides*. *Asian Pac. J. Trop. Med.* **2014**, *7s1*, S491-S496, doi:10.1016/s1995-7645(14)60280-7.
45. Alipour-Gougeh, S.; Asgarpanah, J. Essential and fixed oil chemical compositions of the seeds from the endemic species *Salvia sharifii* Rech. F. & Esfand. *J. Chil. Chem. Soc* **2015**, *60*, 2695-2697, doi:10.4067/S0717-97072015000400012.
46. Samadi, S.; fattahi, B. Study of Compounds of essential oil diversity of some populations of *Salvia staminea* Monthr et Auch. ex Benth in Iran. *TACL* **2014**, *4*, 50-56, doi:10.1080/22297928.2013.770673.
47. Sonmez, U.; Topcu, G.; Ulubelen, A. Constituents of *Salvia verticillata*. *Turk. J. Chem.* **1997**, *21*, 376-382.
